# Effects of Proglumide with Chemotherapy on the Pancreatic Tumor Microenvironment: Phase 1 PROGEM Trial

**DOI:** 10.3390/pharmaceutics18030379

**Published:** 2026-03-19

**Authors:** Jill P. Smith, Gakiza C. Nkulikiyimana, Hong Cao, Wenqiang Chen, Bhaskar Kallakury, John Kwagyan, Anju Duttargi, Benjamin A. Weinberg

**Affiliations:** 1 Department of Medicine, Georgetown University Medical Center, Washington, DC 20007, USA; 2 Department of Oncology, Georgetown University Medical Center, Washington, DC 20007, USAbenjamin.a.weinberg@gunet.georgetown.edu (B.A.W.); 3 Department of Biochemistry, Molecular & Cellular Biology, Georgetown University, Washington, DC 20007, USA; 4 Department of Pathology, MedStar Georgetown University Hospital, Washington, DC 20007, USA; 5 Department of Statistics, Howard University College of Medicine, Washington, DC 20059, USA

**Keywords:** pancreatic ductal adenocarcinoma, fibrosis, tumor microenvironment, proglumide, chemotherapy and combination therapy

## Abstract

**Background**: The primary aim of this Phase 1 clinical trial was to study the safety and dose of a cholecystokinin receptor antagonist, proglumide, in combination with gemcitabine/nab-paclitaxel (GEM-NAB-P) in patients with metastatic pancreatic cancer. The secondary aim was to study the effects of proglumide with GEM-NAB-P on the tumor microenvironment (TME) with tumor biopsies and a blood biomarker assay. An exploratory aim studied the effects of proglumide treatment on cancer-related pain. **Methods**: Gemcitabine-naïve patients were treated with GEM-NAB-P plus proglumide 1200 mg/day. Tumor biopsies and a liquid biopsy serum sample for analysis of a microRNA biomarker panel were collected pre- and on-treatment to study the TME. McGill pain surveys were done at baseline, week 8 and at the end of treatment. The study was approved and registered (NCT05827055). **Results**: The mean age of the patients was 68.2 years (range 54–74 years). The starting dose was well-tolerated with no unexpected treatment-related adverse events observed. Multiplex immunohistochemical analysis of tumor biopsies at baseline and week 8 revealed a significant reduction in Ki67+ cells, collagen1α1, and M2-polarized tumor-associated macrophages (TAMs). Week 8 tumor biopsies demonstrated a significant increase in CD8+ T-cells and natural killer cells compared to baseline. The blood biomarker panel showed a significant inverse change in microRNAs associated with decreasing fibrosis and metastasis. The McGill pain scores showed less pain at week 24 or end-of-treatment compared to baseline. **Conclusions**: Proglumide demonstrates a favorable safety profile when combined with standard chemotherapy for metastatic pancreatic cancer. Its unique ability to remodel TME and alleviate cancer-related pain highlights its potential, warranting further research.

## 1. Introduction

Pancreatic ductal adenocarcinoma (PDAC) has a dismal prognosis [1,2], and the 5-year survival is only about 13% [3]. Even with best front-line chemotherapy regimens, FOLFIRINOX [4] and NALIRIFOX [5], the median survival is still less than one year. The most frequently used chemotherapy for 2nd-line treatment of PDAC is gemcitabine with nab-paclitaxel (GEM-NAB-P) [6] and this regimen is also approved for first-line therapy since it is better tolerated in the elderly [7]. A recent review explored the combination of seven independent agents in combination with GEM-NAB-P for safety and efficacy in previously untreated patients with metastatic PDAC as first-line therapy [8]. The authors concluded that triple regimens with GEM-NAB-P and a third drug may be emerging as an option for first-line therapy in this population.

One reason for the poor response of this cancer is that PDAC is considered a “cold tumor” and lacks CD8+ tumor-infiltrating lymphocytes (TILs). Therefore, immune checkpoint antibodies have largely failed in PDAC [9]. The dense fibrosis of PDAC’s tumor microenvironment (TME) is another reason why immune checkpoint antibodies and chemotherapeutic agents are less effective in this cancer type [10]. Recent studies suggest that compounds that remodel the TME may increase the effectiveness of immunotherapy in PDAC [11]. Remodeling the PDAC TME enhances therapeutic efficacy by overcoming the dense desmoplastic stroma, which otherwise inhibits the infiltration of cytotoxic T lymphocytes and restricts the penetration of therapeutic agents [12,13].

The incidence of PDAC has been increasing [14,15] and there is a disproportionate rise among younger patients [16,17]. Obesity has been reported to more than double the risk for PDAC [18,19]. The prevalence of pancreatic cancer is also increased in countries that consume diets high in saturated fat [20,21,22,23], which stimulates the release of cholecystokinin (CCK) [24] and raises blood levels in humans [25]. Dietary fat was shown to stimulate the growth of tumors in a murine model bearing xenografted pancreatic tumors [26], and the effect was blocked by concomitant treatment with a CCK receptor antagonist, proglumide. We identified that CCK-B receptors (CCK-BRs) are absent in the normal pancreas but emerge during high-fat diet consumption, inflammation, or oncogenic Kras signaling [27], with high expression levels found in PDAC [28].

CCK-BRs have also been identified in both tissue fibroblasts [29] and pancreatic stellate cells (PSCs) [30]. Upon stimulation, these PSCs activate and produce collagen, which is the primary component of fibrosis in pancreatic cancer and chronic pancreatitis [31,32]. Blocking the CCK-BR in activated pancreatic stellate cells (PSCs) induces plasticity, reversing them to a quiescent state, which in turn reduces their pro-fibrotic and tumor-supporting functions [33]. A unique feature we found with proglumide and not with other selective CCK-A or CCK-B receptor antagonists was that proglumide was capable of not only decreasing procollagen production by Western blot in activated pancreatic stellate cells, but it could also degrade established collagen 1α1 [32]. When human pancreatic stellate cells were treated with proglumide and subjected to RNA sequencing to compare differences in molecular pathways, we found that, compared to untreated cells, genes involved in cancer proliferation pathways (*KRAS*, *MYC*, *MET*) were significantly downregulated by proglumide [34]. Genes that are up-regulated when human pancreatic stellate cells were treated with proglumide included the tumor suppressor genes (*TP53*, *CDKN2A*, *SMAD4*, *LATS2*, and *PTEN*) [34]. Genetic editing of the CCK-BR with CRISPR-*Cas*9 technology of activated murine myofibroblasts induces apoptosis and decreases oncogenic signaling [34]. Knockout of the CCK-BR in PSC interrupted cross-communication with cancer epithelial cells, leading to decreased PDAC growth [34].

Combining gemcitabine with proglumide produced a synergistic therapeutic effect in mice with syngeneic pancreatic cancer, outperforming monotherapy with either agent [35]. Histologic analysis in these murine models showed that proglumide treatment alone significantly reduced tumoral fibrosis. This reduction facilitated increased CD8+ T-cell infiltration and a marked rise in intratumoral gemcitabine concentration [35].

A 12-week Phase 1 dose-finding study in 18 patients with metabolic dysfunction-associated steatohepatitis demonstrated that proglumide (800, 1200, and 1600 mg/day) was well-tolerated, effectively reducing serum transaminases and improving fibrosis scores via FibroScan [36]. Subjects receiving 1600 mg/day exhibited higher proglumide blood levels compared to other doses; however, this high-dose group did not achieve steady-state concentrations by the 12th week of the study. Given that the current PDAC proposal mandates a minimum 24-week treatment duration, we have incorporated the FDA-recommended intermediate proglumide dose of 1200 mg/day. This study aimed to evaluate the safety of proglumide paired with chemotherapy in patients with metastatic PDAC. Additionally, we sought to validate pre-clinical findings regarding proglumide’s role in remodeling the tumor microenvironment (TME). The novelty of this study is that we are using a cholecystokinin receptor antagonist and not an additional chemotherapeutic agent to change the TME in an effort to improve response in metastatic PDAC.

## 2. Materials and Methods

### 2.1. Setting and Referral Process

Patients were identified and recruited from the Georgetown Lombardi Comprehensive Cancer Center, Washington, DC. Subjects were enrolled from 9 January 2024 to 1 May 2025. The study was approved by the Georgetown University Institutional Review Board and the Georgetown Lombardi Cancer Center Clinical Trials Committee. The protocol was also approved by the FDA under IND#138481. The full protocol is available in the Supplementary Data S1. The trial was registered on the https://clinicaltrials.gov website (NCT05827055) on 11 April 2023. All the patients signed an informed consent document stating that they were aware of the investigational nature of the study. The study was monitored by the Georgetown Lombardi Data and Safety Monitoring Board.

### 2.2. Research Participants and Enrollment Criteria

Eligible patients were at least 18 years of age with confirmed metastatic PDAC with measurable disease by RECIST v1.1. A performance status of 0 or 1 on ECOG (Eastern Cooperative Oncology Group) and an estimated life expectancy of >3 months were required. Patients were included if they had adequate bone marrow reserve and no other significant medical conditions. Laboratory tests for eligibility included: hemoglobin ≥ 9.0 g/dL, absolute neutrophil count (ANC) > 1500 per mm^3^, platelet count ≥ 100,000 per mm^3^, serum bilirubin ≤ 1.5× institutional upper limit of normal (ULN), aspartate aminotransferase (AST) and alanine aminotransferase (ALT) ≤ 2.5× ULN of normal unless liver metastases are present, in which case it must be ≤5× ULN, and a creatinine clearance (CL) > 60 mL/min. Patients were required to be gemcitabine-naïve; but prior therapy with FOLFIRINOX was allowed. Contraception was required for those of child-bearing potential and a pregnancy test was ordered for each visit in females of child-bearing potential.

### 2.3. Study Design and Objectives

This investigation was an open-labeled Phase 1 study in subjects with metastatic PDAC. The primary objective was to determine the safety and tolerability of proglumide in combination with GEM/NAB-P. The secondary objective was to determine the Phase 2 recommended dose. The other objective was to determine the feasibility of obtaining tumor biopsies and a liquid biopsy to study the effects of the therapy on the tumor microenvironment. The exploratory aim was to study the effects of proglumide on cancer-related pain. The duration of the study treatment was planned for 24 weeks.

After obtaining informed consent, subjects were evaluated at a screening visit with a complete history and physical exam; inclusion criteria laboratory tests were performed. At the baseline visit, a tumor biopsy was obtained by computerized tomography guidance, a blood sample was collected for the research biomarker, and the McGill Pain Questionnaire was performed [37]. Tumors were measured radiographically at baseline and every 8 weeks according to RECIST criteria to determine stability or progression. At each visit, laboratory tests were collected for safety analysis; an interim history and physical exam were performed; the adverse events and drug accountability were evaluated; and a new supply of medication was dispensed with each 4-week cycle. After 8 weeks, a repeat tumor biopsy and research blood were collected for the biomarker liquid biopsy. The McGill pain survey was repeated at week 8 and at the end of treatment. Concomitant medications, including the dosing and frequency of narcotic analgesics were recorded at each visit to determine if proglumide lessened pain.

### 2.4. Treatment/Intervention

Patients were treated with standard of care chemotherapy using gemcitabine (GEM 1000 mg/m^2^ IV) and nab-paclitaxel (NAB-P 125 mg/m^2^) given on days 1, 8, and 15 (every 28 days). Proglumide was given orally and is rapidly absorbed within 1 h. We previously performed pharmacokinetic studies on proglumide and showed it was renally excreted and cleared in about 24 h [38]. Proglumide API was manufactured in bulk by GMP standards and >99% purity by COSMA S.p.A. in Milan Italy. The drug was compounded into vegan capsules containing 400 mg by Custom Prescriptions Pharmacy in Lancaster PA and shipped to the investigation pharmacy. The medication was labeled by the investigational pharmacist and dispensed by the study coordinator every 4 weeks. Patients self-administered proglumide by taking 400 mg orally three times daily (TID) (1200 mg daily). A record log and medicine count were performed at each visit. The protocol called for dosing modifications if ≥2 out of 6 patients at the 400 mg TID dose experienced a dose-limiting toxicity (DLT); if so, the study would be repeated with *N* = 6 subjects using 800 mg daily of (400 mg po BID). If 2 or more experience a DLT at 400 mg BID, then the study would be terminated.

### 2.5. Study Safety Assessments

Safety was evaluated at each visit with laboratory tests; an interim history and physical exam; and recording of any adverse events. Adverse events were recorded, as was the level of severity according to the National Cancer Institute’s Common Terminology Criteria for Adverse Events (CTCAE) version 5 [39], and whether the side effect was related to proglumide.

### 2.6. Tumor Tissue Analysis

After 8 weeks, a repeat tumor biopsy was obtained to study the tumor microenvironment. Tumor biopsies were obtained and paraffin embedded, stained with hematoxylin and eosin (H&E) and Masson’s trichrome stain and then reviewed by the pathologist to confirm adequate cancer sampling. Tissue samples were also analyzed for CCK-BR immunoreactivity with CCK-BR antibody (Ab 77077; 1:200 titer; Abcam, Waltham, MA, USA). Sections of 5 μm thickness were cut from FFPE tissue blocks containing pre-treatment and post-treatment samples from pancreatic tumors. The slides were baked at 60°, deparaffinized in xylene, rehydrated, washed in distilled water and incubated with 10% neutral buffered formalin for an additional 20 min to increase tissue-slide retention. Epitope retrieval/microwave treatment for all antibodies was performed by boiling slides in the respective epitope retrieval buffer ER1, pH 6 or ER2, pH 9; AR9961 or AR9640, respectively (Leica Biosystems; Deer Park, IL, USA). Protein blocking was performed using antibody diluent/blocking buffer (Akoya, ARD1001EA) for 10 min at room temperature. Primary antibody/OPAL dye pairings, staining order and incubation conditions are listed in Table 1 below. Tissue samples were stained using a Leica Bond Autostainer and Phenoimager fusion (Akoya Biosciences, Marlborough, MA, USA) for capturing images, which allows for phenotyping and quantifying tumor–immune cell interactions in the TME. The immunohistochemistry instrument has Multispectral Imaging (MSI) Technology, which allows for easy detection and the measurement of multiple overlapping biomarkers within a single tissue without the interference of autofluorescence and fluorophore crosstalk. Tumor biopsies were reacted with selective polyclonal rabbit anti-human antibodies with the details and dilutions described in Table 1.

### 2.7. Blood Biomarker Panel

Blood serum samples were mixed at a ratio of 1:5 with QIAzol lysis reagent (Qiagen, Cat# 79306; Germantown, MD, USA) and vortexed. The lysate was then extracted with CHCl_3_ and the aqueous phase was further processed for total RNA using the miRNeasy Serum/Plasma Kit (Qiagen, Cat#217184). miRNA was reversely transcribed to cDNA using miRCURY LNA RT kit (Qiagen, Cat#339340). miRNA expression profiling for specific miRNAs (Table 2) was performed using miScript primer assays (Qiagen, Cat#339306) and miRCURY LNA SYBR Green PCR Kit (Qiagen, Cat#339345) in an Applied Biosystems 7300 thermal cycler with the following conditions: initial incubation for 10 min at 95 °C followed by 40 cycles of 95 °C × 30 s, 60 °C × 1 min, and 72 °C for 30 s. A dissociation curve analysis of PCR products was carried out to confirm the specificity of amplification. Data were normalized using hsa-miR-16-5p as an endogenous control. The relative differences between two groups were calculated using the ∆∆CT method.

### 2.8. McGill Pain Questionnaire

The McGill Pain Questionnaire (MPQ) [37] was administered to patients at baseline, week-8 and at the end of treatment. This survey consists primarily of 3 major classes of word descriptors—sensory, affective and evaluative—which are used by patients to specify subjective pain experience. It also contains an intensity scale and other items to determine the properties of pain experience. The MPQ is the most frequently used questionnaire for the multidimensional assessment of pain. The questionnaire was designed to provide quantitative measures of pain that can be treated statistically. The MPQ assesses three separate components of the pain experience: the sensory intensity, the emotional impact, and the cognitive evaluation of pain. The MPQ was recently reviewed [40] and found to hold its strength and reproducibility. There are four strengths of the MPQ. First, the MPQ is a measure of the multiple components (sensory, affective, cognitive, and behavioral) of cancer-related pain, including the nociceptive and neuropathic components of the sensory pain dimension. Second, it allows investigators to collect both quantitative and qualitative data for analysis. Third, the MPQ has good construct, content, and criterion validity, strong reliability for measuring cancer-related pain, sensitivity to treatment effect, and sensitivity to intervention effect. Fourth, the MPQ can be used in many cultures and languages to which it has been translated.

### 2.9. Statistical Evaluation

Immunoreactive cells from the tumor biopsies were counted using Phenochart software (version 2.2.0) in a blinded fashion. Approximately 6–8 images from each biopsy were taken and the mean values from each biopsy at baseline were compared to the mean values from the week-8 biopsies. Analysis comparing the baseline and on-treatment biopsies were done using Student’s *t*-test, since the data assumed a normal distribution. Fibrosis analysis of the collagen content was analyzed by a computer program using ImageJ version 1.54. Graphics were done with GraphPad Prism version 10.

Real-time PCR results of the microRNAs were analyzed using Student’s *t*-test on the normalized mean ΔΔCT (the difference between the cycle counts of the gene of interest minus the count of an endogenous control) values for each group, with Bonferroni corrections applied to adjust for multiple comparisons.

The Generalized Estimating Equations (GEEs) was used to evaluate longitudinal changes in the MPQ outcome measures across the three time points (week 0, week 8, and week 24). Parameter estimates were obtained using Type II Wald chi-square tests to assess the significance of the main effects. All analyses were performed using SPSS version 18.0, with statistical significance set at *p* < 0.05.

## 3. Results

### 3.1. Patient Demographics

Of the eight patients recruited, six were treated, while two were excluded due to screen failures (subjects 003 and 007). There were four males and two females ranging in age from 54 to 74 years of age with a mean age of 68.2 ± 3 years. Of the subjects, three were white non-Hispanic, two were black, and one was Asian. Three had previously progressed after receiving FOLFIRINOX, one had progressed after FOLFIRINOX and a Kras-inhibitor, and three were treatment-naïve. Two patients had metastatic disease in the liver, two had evidence of both liver and peritoneal metastases, and two had metastases in the lungs.

### 3.2. Clinical Outcomes

There were no adverse events (AEs) related to proglumide therapy, and proglumide did not increase the incidence of reported side effects from the chemotherapy. All of the AE(s) reported during the study are shown in Supplementary data Table S1. The adverse events were reviewed by the investigators and the Data Safety Monitoring Committee at the Lombardi Comprehensive Cancer Center to determine if the event was due to the chemotherapy, the disease itself, or to proglumide. None of these AEs were determined to be due to the investigational drug, but instead, they were attributed to the chemotherapy or to disease progression. There were no subject deaths during treatment, but deaths were reported during the survival follow-up interval. There were two protocol deviations due to being 1–2 days outside the window for appointments due to weather. All subjects tolerated the 1200 mg/day dose of proglumide and dose reductions were not required. Although the study was originally planned for 24 weeks, three patients had stable disease and elected to continue on therapy beyond the 24 weeks with IRB approval. One patient with stable disease continued on proglumide and standard of care GEM-NAB-P for 40 weeks before progressing and changing to another research treatment and is still alive at the time of writing this report. The response to therapy according to the RECIST criteria for each patient is shown in Supplementary Figure S1.

### 3.3. Tumor Analysis

**Figure 1:**
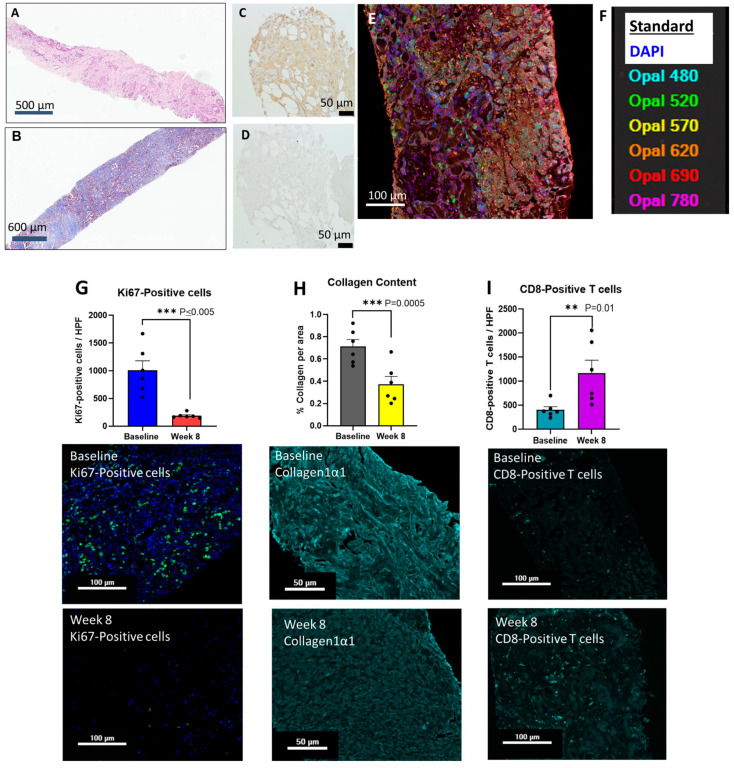

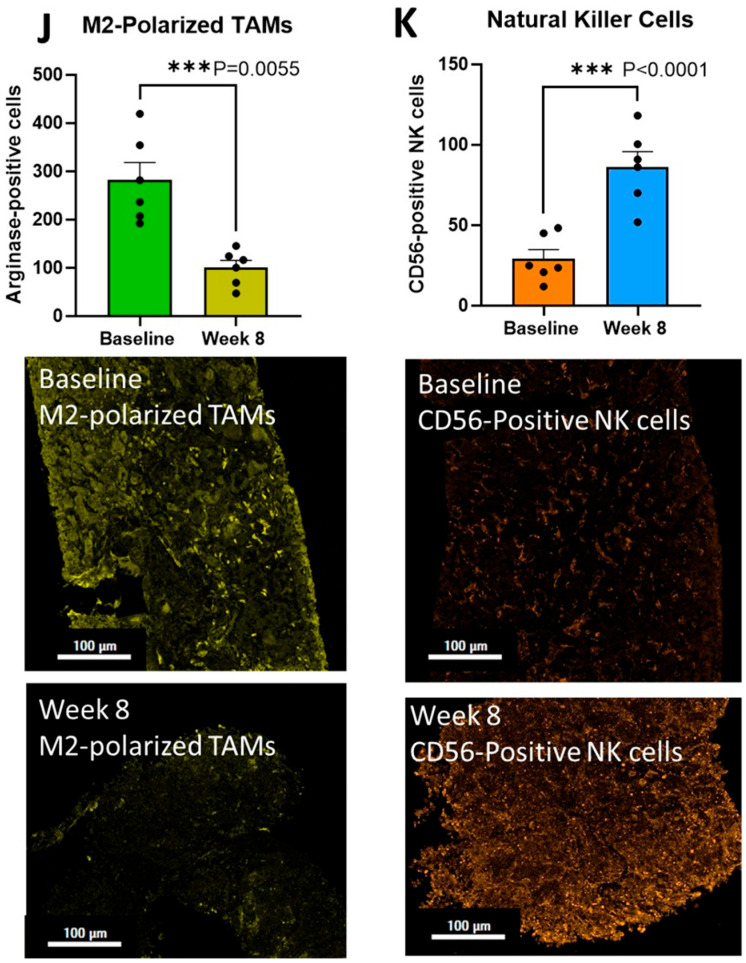
Histology and immunohistochemistry of tumor biopsies. (**A**) Hematoxylin and eosin stain of a tumor biopsy is shown. (**B**) Masson’s trichrome stain of a tumor biopsy is shown. (**C**) Representative tumor stained with the CCK-B receptor antibody. (**D**) Same tumor as panel C showing negative control stained with secondary antibody only. (**E**) Image taken from Phenochart software of a baseline tumor biopsy reacted with the multiplex antibodies for immunohistochemistry showing all the channels. (**F**) Antibody color legend for the Phenochart image shown in panel (**E**). (**G**) Quantification of Ki67 staining of tumor samples at baseline and week 8. (**H**) Analysis of collagen content of tumor biopsies as baseline and week 8. (**I**) The mean number of CD8+ T-cells in tumor biopsies is shown at baseline and week 8. (**J**) The mean number of tumor-associated macrophages (TAMs) is shown in tumors at baseline and week 8. (**K**) Quantification of natural killer (NK) cells in tumors at baseline and week 8 is shown. Beneath each graph is a representative image from a tumor at baseline and week 8 stained with the respective antibody. Approximately 6–8 images from each biopsy were taken and the mean values from each biopsy at baseline were compared to the mean values from the week-8 biopsies. Significant differences include ** *p* < 0.01 and *** *p* ≤ 0.005.

All tumor biopsies were stained with H&E and Masson’s trichrome and reviewed by the pathologist to confirm the presence of cancer. A representative H&E-stained tumor biopsy is shown in Figure 1A. Extensive fibrosis of the tumor microenvironment that is characteristic of pancreatic cancer is easily seen with the H&E stain and more pronounced in the representative image from the Masson’s trichrome stain (Figure 1B). Pre-treatment biopsies were analyzed for CCK-BR immunoreactivity and all of the tissues demonstrated CCK-BRs. A representative pancreatic tumor specimen showing positive staining for the CCK-BR is shown in Figure 1C with the corresponding negative control (Figure 1D). A representative baseline tumor biopsy with all the immunoreactive channels open after staining with the antibodies is shown in Figure 1E and the Phenochart color legend is displayed in Figure 1F. Analysis of the individual immunoreactive channels with representative images at baseline and week 8 are shown in Figure 1G-K. The lognormal *t*-test *p*-values and Ratio of the Geometric Mean (RGM) at 95% confidence intervals (CIs) for each analysis are described below. The Ki67 proliferative marker showed an abundance of immunoreactive cells at baseline, and this number decreased by 81% at week 8 (Figure 1G; RGM = 0.197, 95%CI; 0.125 to 0.31 *p* < 0.005). Extensive tumoral fibrosis, as demonstrated in the Masson’s trichrome stain, was confirmed by measuring collagen immunoreactivity in the biopsy specimens. The collagen content decreased by 48% at week 8 compared to baseline values (Figure 1H; RGM = 0.49, 95%CI; 0.30 to 0.79; *p* = 0.0005). With the decrease in tumoral fibrosis, there was a parallel influx of CD8+ T-cells that increased in the week-8 biopsies by 290% over baseline values (Figure 1I; RGM = 2.685, 95%CI; 1.427 to 5.055; *p* = 0.01). M2-polarized TAMs were abundant in baseline pancreatic tumor biopsies, and these immunosuppressive cells decreased in number by 64% in the week-8 biopsies (Figure 1J; RGM = 0.35, 95%CI; 0.21 to 0.56; *p* = 0.0055). Natural killer cells (NK cells) increased by 197% in tumor biopsies at week 8 compared to baseline (Figure 1K; RGM = 3.21, 95%CI; 1.87 to 5.52; *p* < 0.0001). Representative baseline images and corresponding week-8 images are shown in each figure below the quantitative analysis.

### 3.4. Liquid Biopsy, Blood Biomarker Panel

**Figure 2:**
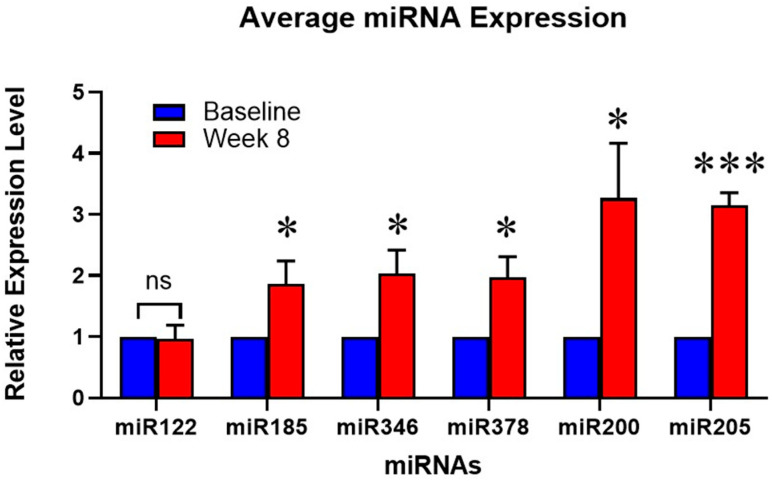
Blood biomarker panel. Columns represent the mean relative values from all six participants that were analyzed in triplicate by PCR for the measurement of serum microRNA at week 8 compared to baseline. miR122 correlates with tumor growth and cell cycle. miR185, miR346, and miR378 measure the inverse relationship to tumor fibrosis or the inhibition of fibrosis. miR200 and miR205 inhibit epithelial-to-mesenchymal transition and the risk for metastases. ns = not significant, * *p* < 0.05 and *** *p* < 0.005.

The mean relative microRNA blood biomarkers expression for the patients at baseline and week 8 are shown in Figure 2. As a group, miR122 did not significantly change at week 8 compared to baseline; however, this miRNA increased in subject #008 and correlated with the progression of the disease. The three miRNAs that are associated with the inhibition of fibroblast or stellate cell activity and fibrosis (miR185, miR346, and miR378) all increased at week 8 compared to baseline values, implying that the tumor-associated fibrosis was decreasing. miR200 and miR205 were significantly increased at week 8 compared to baseline, suggesting a decrease in EMT and metastatic potential. The increase in the “anti-EMT” biomarkers (miR200 and miR205) correlated with the decrease in patients’ target lesions measured radiographically by RECIST criteria at week 8. Overall, the liquid biopsy miRNA biomarker panel correlated with the clinical course of disease and with the tumor biopsies.

### 3.5. McGill Pain Questionnaires

**Figure 3:**
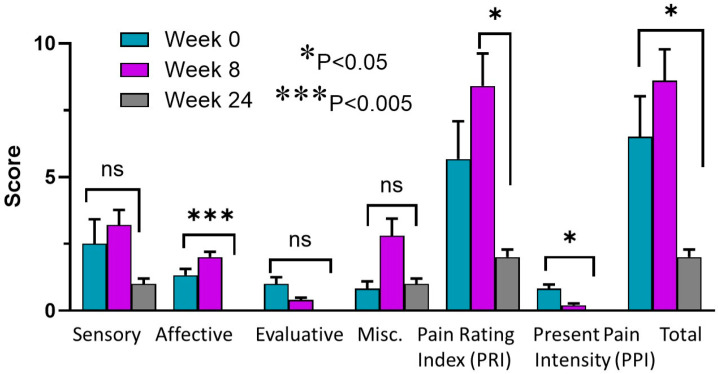
McGill pain scores for each category and total score. Columns represent the mean scores of all patients at baseline (week 0) and week 8 or 24. No difference was observed at week 8 compared to baseline but significant improved pain was observed at week 24 or at end-of-treatment compared to baseline or compared to week 8 (for PRI). * *p* < 0.05; *** *p* < 0.005.

One subject was taking tramadol prn for pain and subject was taking morphine 15 mg BID. The others were not on narcotics and proglumide reduced pain in some categories using the McGill pain survey compared to baseline (Figure 3). We did not observe significant reduction in pain at week 8 compared to baseline in the patients; however, there was a significant improvement at week 24 or at the end of treatment (EOT) in pain surveys. Significant changes were noted in the following categories: affective, pain rating index (PRI), present pain intensity, and the total score, as shown in Figure 3.

We applied the Generalized Estimating Equations (GEEs) to analyze changes in three time points (Week 0, 8, and 24). The overall time effect of total score was statistically significant (Wald χ^2^ = 6.593, *p* = 0.037), suggesting a time-dependent trend. Pairwise comparisons revealed a significant reduction in total score between week 8 and week 24 (B = 6.600, *p* = 0.022), indicating improvement over time, where B represents the estimated mean difference in outcome scores between time points. The overall time effect of affective score was statistically significant (Wald χ^2^ = 18.708, *p* = 0.0001), also suggesting a time-dependent trend. Pairwise comparisons revealed a significant reduction in affective score between week 0 and week 24 (B = 1.333, *p* = 0.017), and between week 8 and week 24 (B = 2.000, *p* = 0.0001), indicating improvement over time. Pairwise comparisons revealed a significant reduction in the pain rating index (PRI) between week 8 and week 24 (B = 6.400, *p* = 0.032). The overall time effect of present pain intensity (PPI) was statistically significant (Wald χ^2^ = 8.793, *p* = 0.012), suggesting a time-dependent trend.

## 4. Discussion

This Phase 1 clinical trial is the first human study to examine the safety of proglumide in combination with chemotherapy. The investigational drug demonstrated a favorable safety profile, with the 1200 mg daily dose being well-tolerated and requiring no dose adjustments. Although not designed to measure efficacy, this study observed stable disease in 50% of the participants after 24 weeks, prompting continued treatment. Additionally, RECIST-defined responses were observed in four out of six patients. The median progression-free survival (PFS) for patients with metastatic pancreatic cancer is generally low, typically ranging between 3.8 and 6.4 months [6,41]. The goal of this Phase 1 clinical trial was not to determine PFS; PFS is better analyzed in a Phase 2 clinical trial with the appropriate controls.

An important finding of this investigation was that proglumide, in combination with chemotherapy, induced favorable remodeling of the tumor microenvironment (TME) in patients with metastatic PDAC. The TME of PDAC is complex, and pancreatic cancer is considered a “cold” tumor. Over the past decade, strategies have been developed to tackle one compartment or another of the pancreatic TME unsuccessfully. Proglumide offers a novel approach in that it provides a two-for-one or even three-for-one targeted approach. The three compartments it effectively alters are (1) the cancer epithelial cell, (2) the fibroblast, and (3) the immune cell compartment. Because our pre-clinical models demonstrated that proglumide, and not chemotherapy, reduced tumoral fibrosis and modulated immune cells (CD8+ T-cells/M2-polarized TAMs), we conclude that the changes observed in patient biopsies at week 8 were most likely driven by the addition of proglumide. However, since patients were also receiving the combination of gemcitabine and nab-paclitaxel in this Phase 1 trial, conclusive results cannot be drawn from these data until a randomized placebo-controlled trial is performed. Increased CD8 T-cell infiltration indicates a robust anti-tumor immune response associated with better clinical prognosis. Reduced Ki67-positive cells in week-8 biopsies indicate lower tumor proliferation, while decreased Arginase 1-positive M2 macrophages signify diminished immunosuppression.

Longitudinal analysis of the blood microRNA panel revealed changes that correlated with histological and clinical outcomes. Although the microRNA biomarker panel was an exploratory aim, the results suggest that it may serve as a promising non-invasive biomarker for TME modulation in the future. miR122 regulates cell cycle [42] and a decrease is consistent with slowed growth; miR185, miR346, and miR378 inhibit collagen/fibrosis [43,44,45,46], and miR200 and miR205 [47,48] inhibit epithelial-to-mesenchymal transition (EMT) and metastases. An increase in these miRNAs indicates decreased tumor fibrosis and metastasis. Rather than selecting miRNAs randomly or from the literature, we developed this panel based on a comprehensive 364-miRNA analysis of pancreatic tissues from proglumide-treated and untreated Kras mice, focusing on differentially expressed miRNAs [49]. Based on these data from the Kras mouse, we analyzed key microRNAs circulating in the blood of mice treated with gemcitabine, with or without proglumide [35], focusing only on those detectable in circulation. Several microRNAs that predicted reduced pancreatic fibrosis in mice were tested in our Phase 1 dose-finding study in liver patients. In this prior liver trial, we found that elevated levels of these microRNAs—indicating fibrosis degradation or stellate cell quiescence—correlated with reduced liver fibrosis scores on FibroScan. In the current investigation, we demonstrated that the expression of fibrosis-inhibiting microRNAs (miR-185, miR-346, and miR-378) increased in PDAC subjects, correlating with a reduction in collagen within the tumor biopsies [43,46,50]. A decline in miR-122-5p is indicative of decreased growth of pancreatic cancer [42]. Within the primary tumor, miRNAs have been shown to regulate EMT by phenotypic assays and by direct targets involved in the EMT pathway. Recently, the five-member *miR-200* family (*miR-141*, *-200a*, *-200b*, *-200c*, and *-429*) and *miR-205* have been identified as EMT-suppressive or “tumor-suppressive” miRNAs directly targeting *ZEB1* and *ZEB2* [51,52,53]. The *miR-200*–ZEB1–E-cadherin axis has been clarified to be a crucial pathway downstream of TGF-β in EMT while reciprocal repression between *ZEB1* and the *miR-200* family has recently been reported to promote EMT and invasion in cancer cells [51,54,55].

Since CCK receptor blockade has been shown to improve pain and decrease the use of narcotic analgesics [56,57], we included the McGill’s pain survey [37] to study the role of proglumide on cancer pain. Previous studies have shown that proglumide enhances morphine-induced analgesia across systemic, intrathecal, and intracerebral routes [58]. Proglumide was first recorded to have selective antagonism of excitatory effects of cholecystokinin in the central nervous system in 1983 [59]. In a prior chronic pancreatitis study [60], oral proglumide monotherapy did not decrease pain in 8 weeks; however, improvements in chronic pancreatitis pain were observed after 12 weeks. Similar to the prior chronic pancreatitis investigation, a reduction in pain was not observed in the current PDAC study at week 8 but was significantly decreased by week 24 or at the end of treatment. Pain reduction may be driven by two potential mechanisms: blocking the excitatory effects of cholecystokinin within the central nervous system or reducing nerve inflammation in the peri-pancreatic region [59]. Another mechanism resulting in decreased pain with proglumide could involve crosstalk between G-protein-coupled receptors (GPCRs). These receptors are known to “cross-talk” or influence the action of other GPCRs, either by sensitizing or desensitizing the intracellular signaling or downstream pathways of each other or by forming heterodimers [61] to mediate physiologic effects. Since both CCK and opioid receptors are G-protein-coupled receptors (GPCRs), blocking CCK receptors with proglumide can prevent opioid receptor desensitization, thereby reducing pain perception.

Limitations to this study include the small sample size and the open-labeled design of the clinical trial. Although this approach is considered acceptable for Phase 1 studies for safety and dosing, additional conclusions should be drawn with more patients and with a chemotherapy monotherapy treatment arm for a control. Another possible limitation is the small biopsy size that restricted the ability to perform other evaluations on the tissues such as RNA expression and gemcitabine uptake, as we previously described in animal models [35]. Our eligibility criteria included patients that were gemcitabine-naïve; however, half of the patients had progressed on front-line FOLFIRINOX and one had also failed treatment with a Kras-inhibitor. Hence, the patients’ prior therapies were heterogeneous. In the proposed Phase 2 clinical trial, we plan to only enroll those eligible to received GEM-NAB-P as a second-line therapy.

New Kras-inhibitor drugs are being tested in patients with pancreatic cancer [62]. However, the therapeutic use of new Kras-inhibitors is frequently limited by the development of resistance, as cancer cells rewire to utilize alternative growth pathways [63]. The PI3K–AKT–mTOR pathway is a key adaptive mechanism driving Kras resistance, prompting researchers to develop targeted therapies against this alternative signaling route. Given that proglumide inhibits key oncogenic pathways like PI3K–AKT–mTOR [34,49], combining it with Kras-inhibitors in future studies could offer a safe, effective method for reducing treatment resistance. Our previous research demonstrated that proglumide treatment successfully arrested the progression of precancerous PanINs in the Kras mouse model, even with continuous Kras expression [64].

Novel approaches are needed to improve the treatment of recalcitrant cancers, such as PDAC. Although proglumide has been shown to exhibit anti-tumor effects similar to gemcitabine in murine models of PDAC, monotherapy is not an option due to the aggressive nature of PDAC. A meta-analysis was performed to compare combination therapy versus gemcitabine monotherapy and concluded that combination therapy significantly improved overall survival compared to gemcitabine alone [65]. Adjuvant therapy with a modified FOLFIRINOX regimen led to significantly longer survival than gemcitabine among patients with resected pancreatic cancer, at the expense of a higher incidence of toxic effects [66]. The Phase 3 PRODIGE 4/ACCORD 11 trial established FOLFIRINOX as a superior first-line treatment for metastatic pancreatic cancer compared to gemcitabine [67]. A risk of increasing the number of drugs administered to patients, however, includes increasing toxicity. Unfortunately, many compounds tested for pancreatic cancer in the pre-clinical setting fail in the clinic due to the multifactorial complex nature of PDAC [68]. These factors include patient characteristics such as the late diagnosis of PDAC and the older population affected, and the histologic features including the dense desmoplastic fibrosis surrounding the cancer epithelial cells and the increased immunosuppressive M2-polarized TAMs. Proglumide, a CCK receptor antagonist, safely modulates the tumor microenvironment (TME) to enhance the efficacy of companion therapies. Findings from this Phase 1 safety study support advancing proglumide to a Phase 2 trial for patients with metastatic PDAC.

## 5. Conclusions

In this Phase 1 study, the oral cholecystokinin receptor antagonist proglumide (1200 mg/day) demonstrated a safe profile when combined with gemcitabine and nab-paclitaxel for treating metastatic pancreatic cancer. Pre- and on-treatment tumor biopsies demonstrated that proglumide remodels the tumor microenvironment (TME) by decreasing fibrosis and M2-polarized tumor-associated macrophages, while enhancing T-cell infiltration. Changes in a non-invasive blood-based miRNA panel mirrored the changes in tumor histology. Furthermore, a survey measuring cancer-related pain showed improvement after 8 weeks on proglumide. Given its favorable safety profile, anti-tumor/anti-fibrotic properties, and capacity to alter the immune cell signature of pancreatic tumors, proglumide warrants further evaluation in Phase 2 clinical trials.

## 6. Patents

Georgetown University is the owner of an issued patent concerning this work: Patent application #16/493,882 and patent #11,278,551. There is also a continuation patent application #17/678,754 and issued patent #12,453,735.

## Figures and Tables

**Table 1:** Antibody panel used for Multiplex Immunohistochemistry.

	Antibody 1	Antibody 2	Antibody 3	Antibody 4	Antibody 5	Antibody 6
Antigen	CD56	Ki67	Collagen1-α1	Arginase1	panCK	CD8
Company	Abcam	Dako	ThermoFisher	ThermoFisher	Agilent	ThermoFisher
Cat #	Ab133345	M7240	PA5-89281	PA5-29645	Z0622	PA5-79010
OPAL Fluor	620	520	690	570	480	780
Dilution	100	100	100	200	100	100
Incubation time	1 h	30 min	1 h	30 min	30 min	1 h
Incubation temp	RT	RT	RT	RT	RT	RT
Antigen Retrieval	ER1	ER1	ER1	ER2	ER2	ER2

RT = room temperature, ER1 = epitope retrieval solution at low pH, ER2 = epitope retrieval solution at high pH. Dako (Carpinteria, CA, USA); ThermoFisher (Rockville, MD, USA); Agilent Technologies (Santa Clara, CA, USA).

**Table 2:** microRNA primers used for the PCR analysis of blood biomarker assay, their function and predicted change with proglumide.

Hsa-microRNA (Human)	GeneGlobe ID	Cat # (Qiagen)	Function	Expected Change with Proglumide
miR-122-5p	YP00205664	339306	Cell cycle regulation, growth	decrease
miR-185-5p	YP00206037	339306	Inhibits proliferation and induces apoptosis	Increase
miR-346	YP00206009	339306	Inhibits growth, migration, and fibrosis	Increase
miR-378e	YP02103282	339306	Inhibits fibrosis	Increase
miR-200b-5p	YP00204144	339306	Inhibits EMT, blocks metastasis	Increase
miR-205-5p	YP00204487	339306	Inhibits EMT, supports epithelial phenotype	Increase
miR-16-5p	YP00205702	339306	PCR internal normalizer	NA

EMT = epithelial-to-mesenchymal transition; PCR = polymerase chain reaction.

## Data Availability

Due to privacy and proprietary concerns, all de-identified data is contained within the article or supplementary material. Additional study information will be available on the public website www.clinicaltrials.gov after review is complete by the Protocol Registration System (PRS) team. Further inquiries can be directed to the corresponding author.

## Supplementary Materials

The following supporting information can be downloaded at: https://www.mdpi.com/article/10.3390/pharmaceutics18030379/s1, Supplemental data S1: Protocol; Supplementary Table S1: All adverse events reported during the study; none were due to proglumide. Supplementary Figure S1: Individual patient response according to RECIST criteria.

## Abbreviations

The following abbreviations are used in this manuscript:

AEs	adverse events
CCK-BR	cholecystokinin B receptor
CTCAE	Common Terminology Criteria for Adverse Events
EMT	epithelial-to-mesenchymal transition
ER	epitope retrieval
FFPE	Formalin-Fixed Paraffin-Embedded
GEEs	Generalized Estimating Equations
GEM-NAB-P	gemcitabine/nab-paclitaxel
GPCRs	G-protein-coupled receptors
MASH	metabolic dysfunction-associated steatohepatitis
H&E	hematoxylin and eosin
MPQ	McGill Pain Questionnaire
NK	natural killer
PDAC	pancreatic ductal adenocarcinoma
PSCs	pancreatic stellate cells
RT	room temperature
TAMs	tumor-associated macrophages
TID	three times daily
TILs	tumor-infiltrating lymphocytes
TME	tumor microenvironment
ULN	upper limit of normal
