# Supplementary material for: Effects of Proglumide with Chemotherapy on the Pancreatic Tumor Microenvironment: Phase 1 PROGEM Trial
Source: Pharmaceutics. 2026 Mar 19;18(3):379. doi: 10.3390/pharmaceutics18030379 (PMC13029477; doi:10.3390/pharmaceutics18030379)
Supplement: Supplementary file 1 [file pharmaceutics-18-00379-s001.zip › pharmaceutics-4157720-supplementary.pdf]

Supplementary data

Supplemental data S1: Protocol

PROTOCOL TITLE:

Role of cholecystokinin receptor blockade on the tumor microenvironment in pancreatic cancer / Protocol 01899

PRINCIPAL INVESTIGATORS:

Clinical PI: Name: Benjamin A. Weinberg, MD
Email Address: benjamin.a.weinberg@gunet.georgetown.edu
Scientific PI: Jill P. Smith, MD
Email Address: jps261@georgetown.edu
Departments: Medicine and Oncology
Telephone Number: 202-687-7583

VERSION NUMBER/DATE:

Version 6, October 23, 2023

REVISION HISTORY

Revision #	Version Date	Summary of Changes
1 (original)	October 30,2018	
2	May 28, 2019	Changed durvalumab to Gem-NAB
3	Jan 12, 2020	Eliminated Phase 1 study, Dose modified
4	8/27/20	Modified sample size and added FOlFIRINOX
5	2/27/21	Changes for IRB
6	10/23/2023	Minor corrections and typos

Table of Contents

Table of Contents 2-3
1.0 Study Summary 4
2.0 Objectives 5
3.0 Background 5
Summary: 5
3.1 Background literature: 6
3.2 Preliminary data: 10
4.0 Study Endpoints 14
4.1 Primary endpoints: 14
4.2 Secondary endpoints: 14
5.0 Study Intervention/Investigational Agent 14
5.1 Formulation/packaging/storage 15
5.2 Doses and treatment regimens 15
5.3 Product preparation 15
5.4 Dose administration 16
5.5 Accountability and dispensation 16
5.6 Disposition of unused investigational study drug 16
6.0 Assessment of Safety 16
6.1 Safety parameters 16
6.2 Definition of serious adverse events 17
6.3 Dose Limiting Toxicity; 17
7.0 Procedures Involved 18
7.1 Study design: 18
7.1a Schedule of events 20
7.2 Screening phase 23
7.3 Baseline visit 23
7.4 Treatment phase: (Baseline to week 24) 23
7.5 End of treatment 24
7.6 Description of study procedures 24
Medical history and physical examination, weight, and vital signs 24
8.0 Data and Specimen Banking 25
9.0 Sharing of Results with Subjects 25
10.0 Study Timelines 25
11.0 Inclusion and Exclusion Criteria 25
11. 1 Inclusion criteria 25
11.2 Exclusion criteria 27
12.0 Vulnerable Populations 28

13.0 Local Number of Subjects 28
14.0 Recruitment Methods 29
15.0 Withdrawal of Subjects 29
16.0 Risks to Subjects 30
17.0 Potential Benefits to Subjects 30
18.0 Sample size and statistical evaluation 31
18.1. Data accuracy & protocol compliance 32
19.0 Data Management and Confidentiality 32
20.0 Provisions to Monitor the Data to Ensure the Safety of Subjects 34
21.0 Provisions to Protect the Privacy Interests of Subjects 34
22.0 Compensation for Research-Related Injury 35
23.0 Economic Burden to Subjects 35
24.0 Consent Process 35
25.0 Process to Document Consent in Writing 36
26.0 Setting 36
27.0 Resources Available 36
28.0 Multi-Site Research 36
29.0 References 36

GHUCCTS Questions GHUCCTS is the Georgetown-Howard Universities Center for Clinical and Translational Science. **Is this study a GHUCCTS Study? _X___Yes ____No. Is the project being sponsored or funded by GHUCCTS? _X___Yes ____No Does the project utilize GHUCCTS services or facilities? __X__Yes ____No (e.g., is the study conducted on the Clinical Research Unit (CRU), is the study supported by a GHUCCTS biostatistician, etc.)**

Study Summary

Study Title: Proglumide therapy for metastatic pancreatic cancer
Research Intervention(s)/ Investigational Agent(s): Proglumide
IND# 138481
Study population: Metastatic pancreatic cancer

**Part 1: Study Design**	Phase 1, Open labeled, Lead-in trial
**Primary Objectives**	Safety
**Secondary Objective(s)**	RP2D
**Sample Size**	6-9
**Study Duration for individual participants**	24 weeks

**Part 2: Study Design**	Phase 2
**Primary Objectives**	• To determine if proglumide changes the pancreatic tumor microenvironment
**Secondary Objective(s)**	• Overall survival<br>• Progression-free survival<br>• Response rate
**Sample Size**	24
**Study Duration for individual participants**	24 weeks
**Study Specific Abbreviations/ Definitions**	CCK=cholecystokinin<br>GEM=gemcitabine<br>MTD= maximally tolerated dose<br>NAB-P= nab-paclitaxel<br>OS= overall survival<br>PDAC: pancreatic ductal adenocarcinoma<br>TILs: tumor infiltrating lymphocytes<br>TAMs: tumor associated macrophages<br>TME= tumor microenvironment<br>mPDAC = metastatic pancreatic ductal adenocarcinoma

Objectives

1.1 Part 1, Phase 1:

- Primary objective: Safety. Determine the safety and tolerability of proglumide in combination with GEM/NAB-P for patients with mPDAC
- Secondary objective: RP2D

1.2 Part 2, Phase 2:

- Primary objective: Evaluate the effect of proglumide therapy on the pancreatic TME in terms of fibrosis and number of TILs
- Secondary objectives: determine the median overall survival, median progression-free survival, and objective response rate in patients with mPDAC treated with proglumide in combination with GEM/NAB-P

2.3 Exploratory aim; Pain management

- Pain control: An exploratory outcome will include pain assessment and pain surveys will be done. Since CCK receptor blockade has been shown to improve pain and decrease the use of narcotic analgesics (McCleane, 2003; Watkins et al., 1984), we will include the McGill's pain survey (Melzack, 1975). The McGill Pain Questionnaire consists primarily of 3 major classes of word descriptors — sensory, affective and evaluative — that are used by patients to specify subjective pain experience. It also contains an intensity scale and other items to determine the properties of pain experience. The questionnaire was designed to provide quantitative measures of pain that can be treated statistically. Also concomitant medications, including dosing and frequency of narcotic analgesics will be recorded at each visit to determine if proglumide lessens pain

Background

Summary: Metastatic pancreatic ductal adenocarcinoma (mPDAC) has typically been resistant to immunotherapy and refractory to chemotherapy; therefore, novel strategies are needed to enhance therapeutic response. Treatment of cancer is significantly improved when a cancer-specific target or cell surface receptor is identified. PDAC is a complex and diverse malignancy with a dense fibrotic tumor microenvironment (TME) making target-specific therapy challenging. We have identified a G-protein coupled receptor (GPCR) on the cell surface that is over-expressed in human PDAC called the Cholecystokinin-B receptor (CCK-BR), and when stimulated by the ligands gastrin or CCK, increases proliferation of PDAC. Downregulation of CCK receptor expression decreases cancer growth in mouse models. CCK receptors are also found on fibroblasts and pancreatic stellate cells. Activation of these

receptors is responsible for collagen production and the dense fibrotic microenvironment characteristic of PDAC. The third cell-type that possesses CCK receptors is immune cells and CCK receptor blockade has been shown to alter the immune cell signature and cytokine release. We have been developing novel approaches to attack PDAC by targeting the CCK-BR and penetrating the fibrotic TME. We have shown that treatment with CCK-B receptor antagonists inhibit growth of PDAC in mice, decreases tumor fibrosis, increases influx of infiltrating tumor effector lymphocytes (TILs). This dense fibrotic TME that is deficient in cytotoxic T-cells and with increased immunosuppressive T-regulatory cells and M2-phenotype tumor-associated macrophages has been shown to impede penetration of gemcitabine and other cancer agents.

3.1 Background literature:

In spite of the success in diagnosis and treatment of other cancers over the years, only modest improvements in overall survival has occurred with PDAC with up to 9% for 5 year survival (Hidalgo, 2010; Ryan et al., 2014) ; however, survival and is only 3% for patients who present with metastatic disease (Ryan et al., 2014).. A recent review of trends for cancer incidence and survival had estimated that by the year 2020, pancreatic ductal adenocarcinoma (PDAC) will surpass colon and breast cancer to become one of the top two causes of cancer-related deaths in the USA (Rahib et al., 2014). In fact currently, the 5-year survival rate for pancreatic cancer is about 8-9%, the lowest of any cancer (Siegel et al., 2014). The reasons for the poor survival rates reported for pancreatic cancer include both the inability to diagnose this disease and intervene in the early stages and the relative resistance of PDAC to standard chemotherapy and immunotherapy. One reason provided for the resistant nature of pancreatic cancer has been attributed to the dense fibrotic TME associated with this malignancy (Neesse et al., 2011). It has become recognized that the perpetuation of pancreatic carcinogenesis involves interactions or ‘cross-talk’ between the precancerous epithelial cells and other cells such as endothelial, mesenchymal, and immune cells of the extracellular matrix (Quante et al., 2013). The pancreatic stellate cell has been identified as the specific cellular source of the extracellular matrix (ECM) proteins of this microenvironment (Apte et al., 2004; Apte et al., 2013). When the pancreatic stellate cells are stimulated, they produce collagen (Apte et al., 2013) and the result is the dense stroma associated with pancreatic cancer. One factor potentially contributing to therapeutic resistance in pancreatic cancer may be the rigidity of the ECM that compresses blood vessels, leading to reduced perfusion that ultimately impedes the delivery of drugs to neoplastic cells (Feig et al., 2012). The cancer epithelial – stellate cell interactions have been shown to perpetuate the hypoxia –fibrosis cycle that is so prominent in pancreatic cancer (Erkan et al., 2009). PDAC stroma consists of mesenchymal cells such as fibroblasts and pancreatic stellate cells (PSCs); extracellular matrix proteins; peri-tumoral nerve fibers; endothelial cells, and immune cells (Farrow et al., 2008; Korc, 2007). Since the fibrosis creates a hypoxic environment (Erkan et al., 2009; Gatenby and Gillies, 2004) and impedes the penetration of potentially effective chemotherapy agents and cytotoxic T-killer cells, many scientists have turned their attention to attacking the stoma (Fang and Declerck, 2013; Hingorani et al., 2003; Strauss et al., 2015).

The host immune response is the other key factor contributing to the microenvironment and also the aggressive nature of PDAC. Similar to other malignancies, chronic inflammation from pancreatitis can predispose to the development of PDAC (Yadav and Lowenfels, 2013). The immune cells that are so prominent in the microenvironment of developing and advanced PDAC however do not support anti-tumor immunity (Zheng et al., 2013). Instead, these inflammatory components (which include macrophages, neutrophils and mast cells) promote tumor growth and invasion. In fact, one of the hallmarks of cancer is the need for tumors to evade immune destruction (Hanahan and Weinberg, 2011). Cancers, including PDAC, employ many tools to evade or defeat the anti-tumor immune response (Pardoll, 2012; Weiner and Lotze, 2012).

Components of the tumor metabolic milieu have been shown to regulate these responses (Feig et al., 2012; Quante et al., 2013). Research involving the human pancreas has been difficult due to its retroperitoneal location; however, an engineered animal model with mutated $Kras^{G12D}$ and a Pdx promoter has been developed to aid in the study of pancreatic carcinogenesis (Tuveson et al., 2004). With this model, we have learned that pancreatic carcinogenesis develops through the progression of a histologic lesion called pancreatic intraepithelial neoplasia (PanINs) in the presence mutated *KRAS*, inflammation, and cells of the microenvironment. During PanIN progression, there is an influx of tumor-associated macrophages with an M2 phenotype, increased neutrophils with a N2 phenotype, Th2 cells and T-regulatory cells that contribute to further immune evasion (Zheng et al., 2013).

A major breakthrough in cancer therapeutics came with the discovery of immune checkpoint pathways that are often regulated by tumor cells as a mechanism of immune resistance (Leach et al., 1996). Cytotoxic T-lymphocyte-associated antigen 4 (CTLA4) and programmed cell death protein 1 (PD1) antibodies have been developed and have been shown in some cancers such as melanoma to be clinically effective in reversing the immune resistance and increasing response to chemotherapeutic agents (Pardoll, 2012). However, pancreatic cancer is considered a non-immunogenic cancer, and its tumor microenvironment has a predominance of immune suppressing T-regulatory cells and myeloid-derived suppressor cells but a lack of tumor infiltrating effector T-cells (Feig et al., 2012; Vonderheide and Bayne, 2013; Zheng et al., 2013). Unfortunately, clinical trials using immune checkpoint antibodies have thus far largely failed in pancreatic cancer (Brahmer et al., 2012).

The gastrointestinal peptides, cholecystokinin (CCK) (Smith et al., 1990; Smith et al., 1991) and gastrin (Smith et al., 1995), have been shown to stimulate growth of pancreatic cancer. Researchers have used a decapeptide analogue of CCK, cerulein, to accelerate pancreatic carcinogenesis in animal models such as the nitrosamine model (Howatson and Carter, 1985) or in the KRAS transgenic mouse model (Carriere et al., 2009). Gastrin, a related peptide, is found in the fetal pancreas (Bardram et al., 1990; Brand and Fuller, 1988), but levels rapidly decrease to zero after birth in the pancreas and gastrin expression is then only detected in the adult stomach (Tamiolakis et al., 2004). However, gastrin is re-expressed during pancreatic carcinogenesis in early pancreatic intraepithelial neoplasia (PanIN) lesions (Prasad et al., 2005) and is overexpressed in human pancreatic cancer where it regulates growth by an autocrine mechanism (Smith et al., 1998; Smith et al., 1996). Both gastrin and CCK stimulate growth of pancreatic cancer through G-protein coupled CCK receptors (Smith et al., 1993; Smith et al., 1994), and these receptors are markedly over-expressed in cancer. When gastrin is silenced by RNA interference (Matters et al., 2009) pancreatic cancer growth is significantly decreased. In addition, if the CCK receptor is downregulated (Fino et al., 2012), or the receptors are blocked with CCK receptor antagonists (Smith et al., 1994; Smith and Solomon, 2014) pancreatic cancer growth is inhibited.

Three types of CCK receptors have been characterized and sequenced. The CCK-A receptor is the predominant type found in normal rodent pancreas (Wank et al., 1992) whereas the CCK-B receptor variety is the form found in the normal human pancreas (Wank et al., 1994; Weinberg et al., 1997). Of interest, when the rodent pancreas undergoes malignant transformation as a result of azaserine treatment (Longnecker et al., 1980) or under the influence of mutant *KRAS* (Smith et al., 2014), the rodent pancreas expresses de novo the CCK-B receptor phenotype. The third variety of CCK receptor, the CCK-C receptor, is a splice variant of the CCK-B receptor that occurs only in human pancreatic cancer patients with a germline single nucleotide polymorphism (rs1800843) (Smith et al., 2002; Smith et al., 2012). Although human pancreatic cancer may have both CCK-A and CCK-B receptor subtypes (Rai et al., 2016; Weinberg et al., 1997), CCK and

gastrin cancer growth is mediated through the CCK-B receptor type and its splice variant (Smith and Solomon, 2014). A clinical trial using CCK receptor blockade was conducted many years ago to treat human subjects with advanced pancreatic cancer, and unfortunately, this trial failed because a selective CCK-A receptor antagonist was used rather than a CCK-B antagonist (Abbruzzese et al., 1992). A number of receptor specific CCK antagonists have been developed that have high affinity to either the CCK-A or CCK-B receptor and others are under investigation (Berna and Jensen, 2007). L364,718 (devazepide) is a highly potent and selected antagonist to the CCK-A receptor (Chang et al., 1986), the predominant recent variety in mice and in mouse Panc02 pancreas cancer (Matters et al., 2014). Another, yet weaker, antagonist proglumide (Hahne et al., 1981) is a nonselective antagonist and inhibits the actions of peptides at both the CCK-A receptor and the CCK-B receptor.

CCK receptors have also been identified on tissue fibroblasts (Singh et al., 1995) and pancreatic stellate (Berna et al., 2010) cells; and when these receptors are stimulated, the fibroblasts become activated to produce desmoplastic stroma characteristic of the microenvironment of pancreatic cancer (Apte et al., 2004; Apte et al., 2013). Evidence that CCK receptors play a role in the dense fibrosis of the pancreas cancer microenvironment was demonstrated when CCK-receptor blockade with proglumide inhibited fibrosis in the transgenic mutated *KRAS* murine model for pancreatic cancer (Smith et al., 2014).

Apart from its normal physiologic role in digestion, proliferative effects on cancer epithelial cells, and collagen promoting effects from pancreatic stellate cells, CCK-8 also serves as an immunomodulatory peptide. Furthermore, immune cells also have CCK receptors (Zhang et al., 2014) that respond to CCK receptor blockade to change their cytokine expression signature. CCK can influence the action of specific CD4+ T cell subsets by regulating antigen-presenting cell functions. CCK peptide activates CD4+ T cells and T regulatory cells (Treg), and this effect is blocked with CCK-receptor antagonists (Zhang et al., 2014). Zhang and colleagues (Zhang et al., 2011) showed that CCK peptide administration suppressed Th1 phenotype while enhancing Th2 development and cytokine production; CCKR antagonists blocked this effect. Because CCK receptors are found on three components of the pancreatic cancer microenvironment, (pancreatic epithelial cells, tumor associated fibroblasts, and immune cells), use of pharmaceutical agents that block the signaling at this receptor may be useful in improving therapy to pancreatic cancer.

The significance of this research is that it involves a novel approach to the treatment of a devastating disease with a mean survival with standard chemotherapy of approximately 6-8 months. The research plan is an extension of our team's effort over several years of investigation and reflects a true translation from bench research to bedside care with a novel therapy. The study is designed to provide data on the use of a CCK receptor antagonist to reduce fibrosis of the pancreatic cancer microenvironment and improve standard of care first line chemotherapy. Should this protocol reveal promising results, we plan to design a large randomized multicenter trial.

The provocative preliminary findings that sparked our interest in fibrosis were actually found incidentally when we were studying pancreatic carcinogenesis using p48Cre/LSL-Kras^{G12D} (KC) mutant KRAS transgenic mice. This KRAS murine model develops precancerous PanIN lesions, inflammation, and fibrosis starting at about 3-4 months of age. We tested the hypothesis that CCK-receptor blockade would decrease the progression of the precancerous PanIN lesions (which it did), but we also unexpectedly found it reversed fibrosis. In this model the 4 mos old KC mice were treated with water supplemented with the CCK-receptor antagonist proglumide (0.1 mg/mL, or approx. 30 mg/kg/d per mouse) or untreated water and after 4 months, the mice were ethically euthanized and pancreas dissected, stained and analyzed. Mice on the regular

water showed significant fibrosis in their pancreas with the Mason's trichrome stain (Fig 1A). However, we found that the mice on the proglumide therapy had significantly less PanINs, less inflammation, & markedly less tissue fibrosis (Fig 1B) (Smith et al., 2014). Since the tissue studied was from the mouse endogenous pancreas and it was assumed that activated stellate cells that are known to have CCK-Rs (Berna et al., 2010) in this tissue gave rise to the fibrosis.

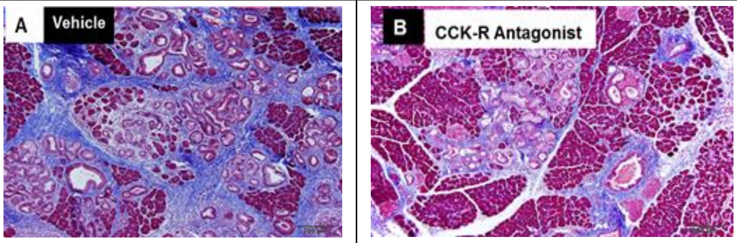

Fig 1. A. Trichrome stain showing extensive fibrosis in an 8 month old KRAS mouse pancreas. **B.** markedly reduced fibrosis in KRAS mice pancreas treated with CCK antagonist (Smith et al., 2014).

In the 2nd experiment we were investigating the role of dietary fat on growth of pancreatic cancer and we used a xenografted murine pancreatic cancer Panc02 cell line injected subcutaneously into immune competent C57BL/6 mice. All mice received a high fat diet and then we treated half of the mice with untreated drinking water (N=10) and another half with water containing proglumide (N=10). Since the pancreatic tumor in this model was remote from the mouse pancreas, the fibrosis that resulted was assumed to originate from tissue-associated fibroblasts (also reported to have CCK-Rs (Singh et al., 1995)). Fig 2A shows marked fibrosis in the murine pancreatic tumor from the control untreated mice. There was a significant reduction in tumor-associated fibrosis in the mice that received the proglumide (Fig 2B). The reduction in fibrosis was scored and was significantly reduced (Fig 3, $p<0.001$). When the stellate cells or fibroblasts are activated, they induce the dense fibrosis characteristic of the micro-environment in pancreatic cancer (Apte et al., 2004; Apte et al., 2013)

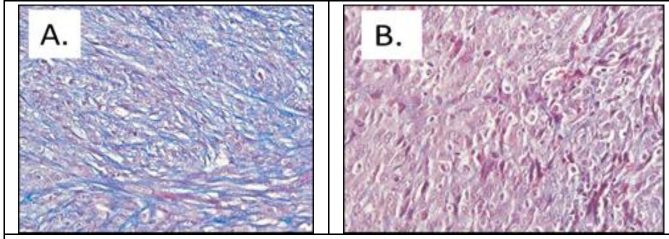

Fig 2. A. Panc02 murine pancreatic tumors show dense blue Masson's trichrome staining of fibrosis. **B.** There is a marked decrease in the fibrosis in mice treated with a CCK-receptor antagonist, proglumide. (Nadella et al., 2018)

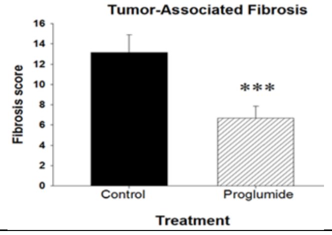

Fig 3. Fibrosis score was elevated in the sc pancreatic tumor was reversed with proglumide (Nadella et al., 2018).

In a 3rd study we demonstrated that proglumide altered the tumor associated fibrosis and also tumor infiltrating lymphocytes rendering pancreatic cancer susceptible to therapy with an immune checkpoint antibody (Smith et al., 2017).

Immune competent C57BL/6 mice bearing pancreatic tumors were treated with PBS (control), proglumide, a PD-1 ab, or the combination of proglumide and the PD-1 antibody. Pancreatic tumors exhibited an influx of CD8+ cells (Fig 4A), a decrease in FoxP3+ (Tregs, Fig 4B), and decreased fibrosis (Fig 5). Tumor sizes were significantly smaller in mice treated with the combination therapy (Fig 6). The combination therapy also resulted in prolonged survival. This study showed proglumide alone decreased fibrosis (Fig 5) and together with the PD-1 ab the tumor infiltrating lymphocytes changed rendering the tumors responsive to therapy.

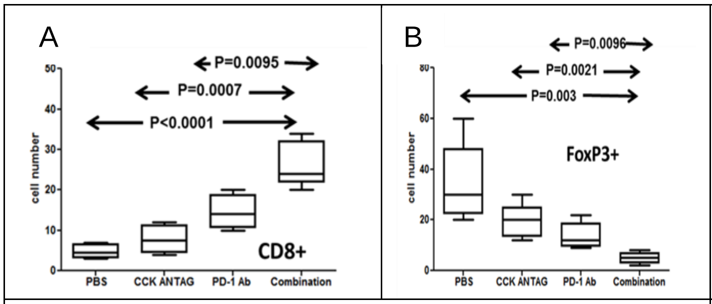	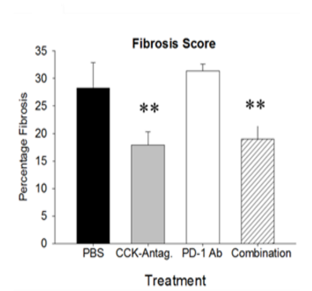	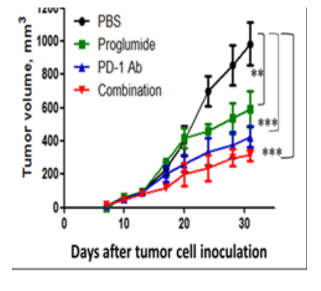
**Fig 4. A.** Lack of CD8+ lymphocytes in PBS treated murine pancreatic tumor. Influx of CD8+ cells occurred with the combination therapy. **B.** FoxP3+ (Tregs) decreased in tumors of mice receiving the combination therapy(Smith et al., 2017).	**Fig 5.** Intratumoral fibrosis was only decreased in mice treated with proglumide. (Smith et al., 2017)	**Fig 6.** Tumor size is significantly decreased with combination therapy. ***p<0.001. (Smith et al., 2017)

3.2 Preliminary data:

In a pilot study (unpublished) in C57BL/6 immune competent mice to determine if proglumide may improve the uptake and efficacy of gemcitabine on murine pancreatic cancer. We injected 500,000 mT3 mouse PDAC cells subcutaneously into each flank of 40 female mice. One week after the mice had measurable tumors (Baseline) they were divided into 4 groups (N=10 mice each) with equal mean tumors volumes in each group. Treatment groups were as follows:

1. PBS control (100ul ip twice weekly)
2. Proglumide in drinking water 0.1 mg/kg
3. Gemcitabine 100mg/kg in 100ul ip twice weekly
4. Combination of gemcitabine & proglumide (same doses as above)

According to the human gemcitabine regimen, we are following the induction schedule of 7 weeks of gemcitabine and week 8 is off (no gemcitabine), followed by 4-week maintenance cycles of 3 weeks with gemcitabine and week-4 no drug. Tumors are measured weekly using a caliper and the volumes calculated with the formula [L x w^2 x 0.5]. According to the IACUC protocol, all mice had to be ethically euthanized when tumors reach 20mm in diameter. Therefore, when tumors reached this size, the mouse was euthanized and tumor removed. The tissue was flash frozen for RNA, protein and mass spectrometry and part placed in 4% paraformaldehyde for histology. We have completed week-17 of the study; which includes the 8-week induction and two cycles of 4-weeks. Tumor volumes for the first eight weeks while all mice were still alive are shown in Fig 7. The slope of the growth curve for the mice treated with both gemcitabine and proglumide is significantly less than PBS controls or either drug as monotherapy (Fig 7).

By day 62, all of the control PBS (control) mice had been euthanized since the tumors reached maximum allowed size. At this same time period (day 62) 3 mice in the proglumide monotherapy-treatment group had died, one mouse in the gemcitabine-only group had died, none in the combination group died. On day 73, with all the PBS control mice still gone, 5 mice in the proglumide group died, 3 in the gemcitabine and one in the combination group.

The Kaplan Meier survival curve (Figure 8) shows that the combination therapy group had significant greater survival compared to controls calculated by Log Rank test. The median survival was best for the combination group followed by gemcitabine, then proglumide, and the PBS control mice exhibited the poorest survival.

Mass spectrometry was performed in the Lombardi Core Proteomics laboratory for measuring drug uptake into tumors. Preliminary analysis shows that the gemcitabine concentration in the monotherapy tumors is a mean of 173 pg/ml and the gemcitabine level in the tumors of mice treated with both drugs is more than four-times the concentration (729 pg/ml). This preliminary data supports our hypothesis that proglumide may improve effectiveness of gemcitabine in treating pancreatic cancer and demonstrates the feasibility of our lab to conduct this study.

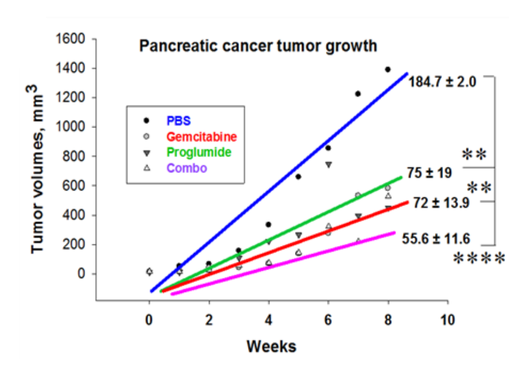

Fig 7. Tumor growth rates over time and slope of growth curve. Statistically significant compared to control (**P <0.006, **** P <0.0001).

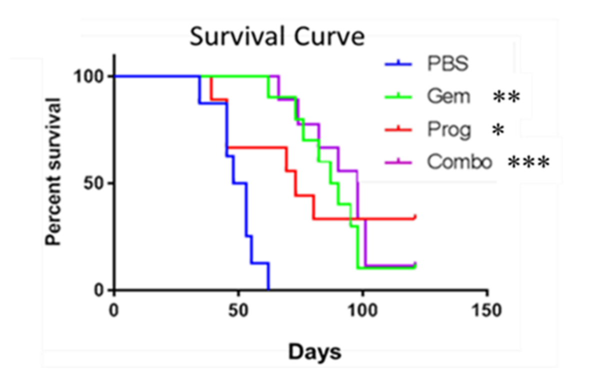

Fig 8. Survival rates of mice treated with proglumide, gemcitabine, or both compared to PBS-treated controls. Log Rank was significant compared to controls; (* P=0.01; **P =0.001, *** P <0.0001).

For analysis of lymphocyte number and fibrosis, multiple images of each slide were captured using a 10X objective lens on an Olympus BX61 microscope with a DP73 camera so that the entire slide was assessed. Images were exported and counted by 2 separate staff in a blinded fashion. Average fibrosis in tumors of all treatment groups as measured from Masson's trichrome staining. Compared to controls, only the tumors of mice treated with proglumide had less fibrosis (Fig 9A &B).

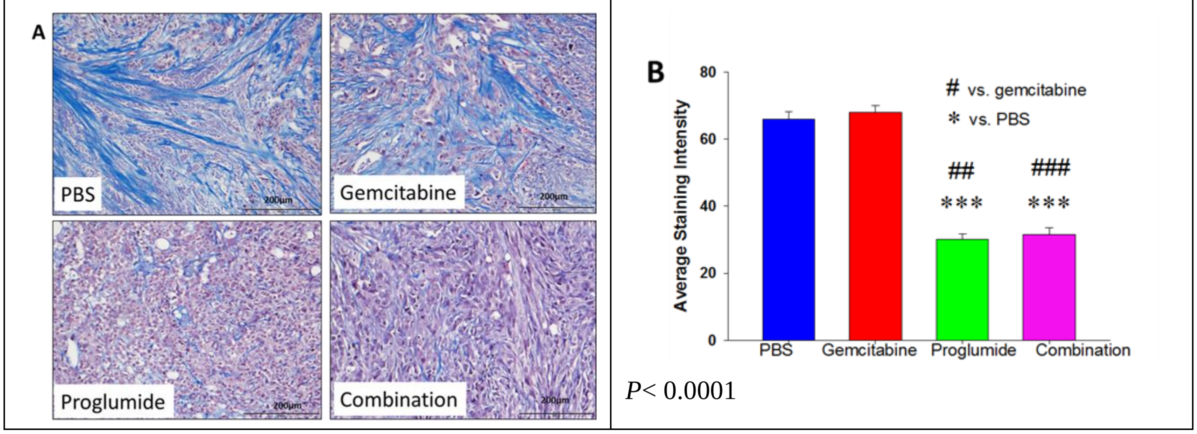

P< 0.0001

**Fig 9A.** Masson's trichrome stain from representative tumor from each group of mT3 tumors.	**Fig 9B.** Computerized analysis shows only proglumide treated mice had less fibrosis in the tumors.

Fixed tumor sections (5 µm) from each group were stained with CD8 Ab (Figs10A &B). Immunoreactive CD8+ cells were counted using ImageJ. Proglumide monotherapy and in combination with gemcitabine therapy increased CD8+ cells significantly. There was no change in CD8+ TILs in gemcitabine monotherapy.

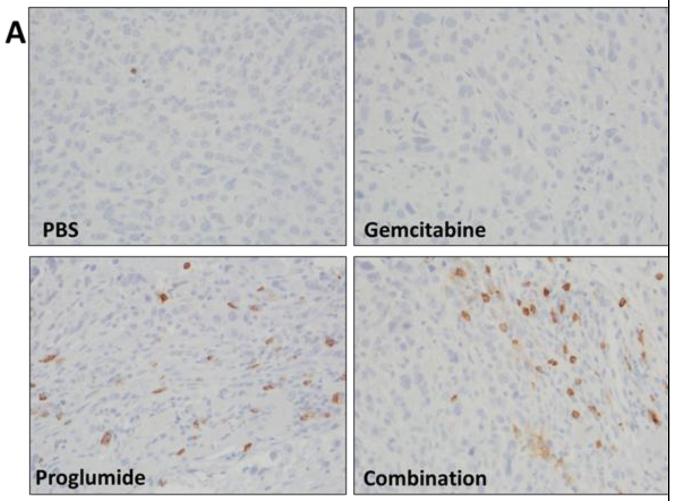

Fig 10A. Representative photos from each group stained for CD8+ lymphocytes.

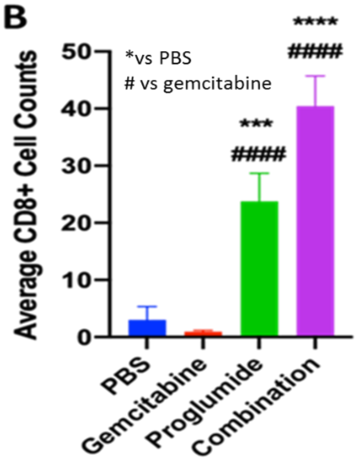

Fig 10B. CD8+ cell number per group. # compared to gemcitabine. Statistical significance: *** P<0.01; **** and #### P<0.0001.

Circulating blood miRNA from mice treated with either Gemcitabine monotherapy or Combination (Gem+Prog) show changes in the miRNAs associated with fibrosis. Since miRNAs are stable in the peripheral blood we will evaluate our panel of miRNA biomarkers as a noninvasive test. Those miRNAs upregulated by proglumide in the pancreas have been shown to inhibit fibrosis, inflammation or both including miR-185-5p, miR-346-5p, miR-378a-3p, and miR708-5p (Table 1).

	GEM	COMBO	
miRNA	Mean ± Std Error	Mean ± Std Error	p value
miR-185-5p	1.31 ± 0.20	2.44 ± 0.37	**0.02
miR-346-5p	2.36 ± 0.58	8.15 ± 0.81	**0.01
miR-378a-3p	1.61 ± 0.37	3.93 ± 0.60	**0.02
miR-708-5p	1.41 ± 0.36	2.58 ± 0.47	**0.001

Table 1. Blood miRNA levels that reflect a change in anti-fibrosis

In a 2nd model of pancreatic cancer, gemcitabine-resistant PANC-1 human pancreatic cancer cells were grown orthotopically in nude mice. The mice received the same treatments as above. The PANC-1 cells were luciferase tagged and treatment was not

initiated until all the mice had detectable tumors. After 6 weeks the mice were ethically euthanized, and tumors were removed and weighed. Tumors of proglumide treated mice were only 14% less in mass compared to PBS controls and this was not significant. Tumors of gemcitabine treated mice weighed 42.5% less than PBS controls (Figure 11; $P<0.05$). Tumors of mice treated with the combination therapy were 59% less in mass that PBS treated tumors ($P=0.0017$) but not significantly different from the mass of the tumors from the gemcitabine-monotherapy treated mice.

One of the most significant findings in this investigation was that the total number of histologically confirmed PANC-1 pancreatic cancer metastases was significantly decreased in the mice with orthotopic tumors treated with the combination of gemcitabine and proglumide (Figure 12). The mean number of metastases identified per mouse was reduced by 89% in mice treated with the combination of gemcitabine and proglumide compared to PBS treated control mice ($P=0.0004$). The total number of metastases counted in PBS mice were 45, whereas the number of metastases in gemcitabine and proglumide treated mice were slightly less at N=30 each. Remarkably, the effect of the combining gemcitabine and proglumide was synergistic on reducing the number of metastases and only N=6 total metastases were counted in this treatment group.

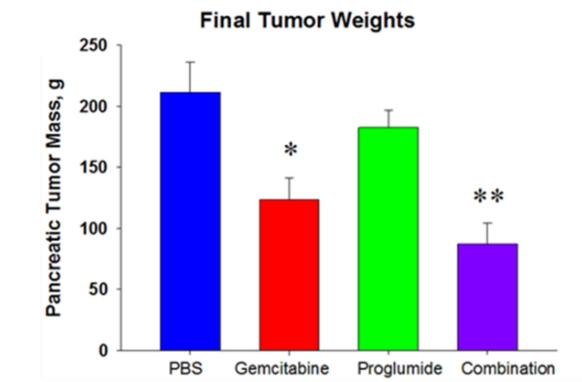

Fig 11. Final tumor weights of PANC-1 human tumors.

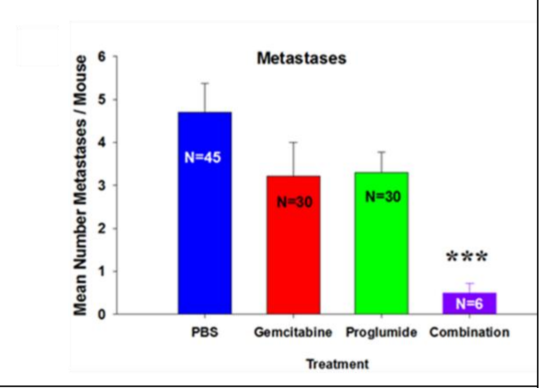

Fig 12. The number of metastases was significantly decreased in the mice treated with both gemcitabine and proglumide.

Significance of findings: Our laboratory is the first to provide evidence indicating a role for CCK receptors and pancreatic neoplasia of humans. We have shown that human pancreatic cancer cells express the CCK-B receptor type predominantly and CCK receptor blockade inhibits cancer growth. We have also shown that CCK receptor down-regulation inhibits cancer growth. In the KRAS animal model CCK receptor blockade halts the progression of precancerous PanIN lesions and reverses the marked fibrosis of the microenvironment. Furthermore, when administered to mice bearing established pancreatic cancers orthotopically or subcutaneously, CCK receptor blockade reverses tumor associated fibrosis and allows for the influx of T-lymphocytes. When combined with immune checkpoint antibodies or gemcitabine, CCK receptor antagonists significantly decrease tumor size and prolong survival, and decrease metastasis. Since CCK receptor antagonists are orally bioavailable and have been already used in human subjects for other indications, their safety and potential toxicity as well as dose has been established. These findings are promising and have raised the possibility concerning

combination therapy of CCK receptor antagonist and chemotherapy or immunotherapy for patients with pancreatic cancer. Since there are no immune checkpoint antibodies approved for first line therapy in pancreatic cancer and because studies have shown chemotherapy may be less effective due to the pancreatic tumor microenvironment, we will initiate our adjuvant therapy with proglumide in human subjects using standard of care chemotherapy. Since proglumide is a small molecule agent rather than a chemotherapeutic compound and because it did not cause any bone marrow or chemical toxicity in the clinical trials, we do not anticipate any increase in the reported toxicity known to occur with chemotherapy.

Study Endpoints

4.1 Primary endpoints:

- The effect of proglumide on the TME in patients with PDAC treated with proglumide compared to patients not treated with proglumide in terms of decrease in fibrosis and increase in TILs

4.2 Secondary endpoints:

- Overall survival
- Progression-free survival
- Objective response rate

Study Intervention/Investigational Agent

Description: Proglumide -Study medication: Proglumide is an older drug that was originally developed for peptic ulcer disease but is no longer marketed in the US. The chemical name for the investigational drug, proglumide, chemically is (RS)-N2-benzoyl-N,N-dipropyl-α-glutamine (Fig 13). It is water soluble with a molecular weight of 334.41 g/mol. It is off patent in the USA and is currently in use in Europe and Asia. Proglumide is a nonselective CCK-A and CCK-B receptor antagonist that is orally bioavailable and has been used in other human clinical trials at daily doses up to 1600 mg/day. Proglumide is a nonselective CCK receptor antagonist meaning it has properties at both the CCK-A and the CCK-B receptor. The rationale for using this compound over more highly selective antagonists for several reasons: stellate cells possess both receptor types,

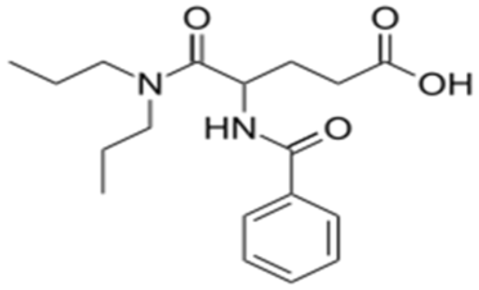

and more potent CCK-A antagonists impair gall bladder emptying (Pauletzki et al., 1995) which may result in acute cholecystitis. Furthermore, CCK-A receptor blockade may improve pain management, and CCK-B blockade may inhibit growth of the PDAC epithelial cells. Proglumide was manufactured in Italy by Rottapharm Biotech and produced as Protaxon forte

Fig 13. Chemical structure of proglumide. CAS: 6620-60-6. Formula: C18H26N2O4, MW: 334.41 g/mol.	(proglumetacin) for pain management. This compound is no longer available.

1.3 *Drug/Device Handling:* The GMP bulk drug will be compounded at the FDA approved Compounding Pharmacy: Custom Prescriptions, LancasterPA under authorized Pharmacist Diane Boomsma, PharmD, FIACP. The drug will be compounded into 300mg vegan capsules and shipped directly to the Investigational Pharmacy at Georgetown University under the direction of Dr. Khang Ho. Prescription will be labeled in the pharmacy at Georgetown University with the name of the investigational compound or placebo, patient name and pharmacy information. The drug will be dispensed by prescription only by one of the investigators and given to the patients by Dr. Weinberg or the Nurse Study coordinator.

1.4 *If the drug is investigational (has an IND) or the device has an IDE or a claim of abbreviated IDE (non-significant risk device), include the following information:*

- *IND 138481*

5.1 Formulation/packaging/storage

Proglumide will be compounded into capsules containing 400 mg of active drug. The drug is stable at room temperature. Capsules will be shipped in bulk to the Investigational Pharmacy and dispensed monthly to participants.

5.2 Doses and treatment regimens

Medication will be labelled by the Investigational pharmacist and dispensed every 4 weeks, enough for the 4-week supply. Subjects will take 400 mg orally TID (1200 mg daily).

5.3 Product preparation

Proglumide will be manufactured by GMP standards and >99% purity by Therefore Proglumide will be manufactured in bulk by the following FDA-inspected company by GMP standards and is approved in the EU:

A.M.S.A. S.p.A. - COSMA S.p.A.
Viale del Ghisallo, 20 - 20151 Milano, Italy
tel. +39-023925326 - fax +39-0239219957

The drug will be compounded by Custom Prescriptions of Lancaster, LLC into capsules containing 400 mg each. Prescriptions will be labeled and dispensed by the investigational pharmacist.

5.4 Dose administration

Proglumide is orally bioavailable and patients will self-administer by mouth.

5.5 Accountability and dispensation

A record log and medicine count will be performed at each visit. Patients will be instructed to bring the prescription container to each visit for drug counting and accountability. Unused drug can be redispensed to the patient. At the end of the study or at withdrawal, all investigational drug will be returned to the investigational pharmacy.

5.6 Disposition of unused investigational study drug

Proglumide will be returned to the Investigational pharmacist and all accountability documents completed. At the end of the study, unused drug will be returned to the Principal Investigator (Jill Smith, MD, Sponsor of the IND) for nonhuman research use.

- *Explain procedures followed to comply with sponsor requirements for FDA regulated research for the following:*

	Applicable to:		
FDA Regulation	***IND Studies***	***IDE studies***	***Abbreviated IDE studies***
21 CFR 11	***X***	***X***	
21 CFR 54	***X***	***X***	
21 CFR 210	***X***		
21 CFR 211	***X***		
21 CFR 312	***X***		
21 CFR 812		***X***	***X***
21 CFR 820		***X***	

Assessment of Safety

The Principal Investigator is responsible for ensuring that all staff involved in the study is familiar with the content of this section.

6.1 Safety parameters

Definition of adverse events

The International Conference on Harmonization (ICH) Guideline for Good Clinical Practice (GCP) E6(R1) defines an AE as:

Any untoward medical occurrence in a patient or clinical investigation patient administered a pharmaceutical product and which does not necessarily have a causal relationship with this treatment. An AE can therefore be any unfavorable and unintended sign (including an

abnormal laboratory finding), symptom, or disease temporally associated with the use of a medicinal product, whether or not considered related to the medicinal product.

An AE includes but is not limited to any clinically significant worsening of a patient's pre-existing condition. An abnormal laboratory finding (including ECG finding) that requires an action or intervention by the investigator, or a finding judged by the investigator to represent a change beyond the range of normal physiologic fluctuation, should be reported as an AE.

Adverse events may be treatment emergent (i.e., occurring after initial receipt of investigational product) or nontreatment emergent. A nontreatment-emergent AE is any new sign or symptom, disease, or other untoward medical event that begins after written informed consent has been obtained but before the patient has received investigational product.

Elective treatment or surgery or preplanned treatment or surgery (that was scheduled prior to the patient being enrolled into the study) for a documented pre-existing condition, that did not worsen from baseline, is not considered an AE (serious or nonserious). An untoward medical event occurring during the prescheduled elective procedure or routinely scheduled treatment should be recorded as an AE or SAE.

The term AE is used to include both serious and non-serious AEs.

6.2 Definition of serious adverse events

A serious adverse event is an AE occurring during any study phase (i.e., screening, run-in, treatment, wash-out, follow-up), at any dose of the study drugs that fulfils one or more of the following criteria:

- Results in death
- Is immediately life-threatening
- Requires in-patient hospitalization or prolongation of existing hospitalization
- Results in persistent or significant disability or incapacity
- Is a congenital abnormality or birth defect in offspring of the patient
- Is an important medical event that may jeopardize the patient or may require medical intervention to prevent one of the outcomes listed above.
- Medical or scientific judgment should be exercised in deciding whether expedited reporting is appropriate in this situation. Examples of medically important events are intensive treatment in an emergency room or at home for allergic bronchospasm, blood dyscrasias, or convulsions that do not result in hospitalizations; or development of drug dependency or drug abuse.

6.3 Dose Limiting Toxicity;

Cytopenias can be anticipated from the Gem-NAB-P regimen.

DLT (Dose limiting toxicity) is defined as (i.e., any Grade ≥ 3 adverse event that it is likely, possibly, or probably related to the investigational drug proglumide unless there is a clear alternative cause) that occurs within 28 days from the first dose of the study drug.

Procedures Involved

1.5 *Provide a description of all research procedures being performed and when they are performed, including procedures being performed to monitor subjects for safety or minimize risks:* The tumor core biopsy is required for research and the tissue will be analyzed ex-vivo for tumor fibrosis by histology and expression of RNA fibrosis genes by qRT-PCR. We will utilize mass cytometry (CyTOF) to facilitate with analysis of small tissue specimens. Tissues will also be evaluated for TILs and TAMs by IHC. A separate consent form will be signed for the tissue biopsy. Risks of a biopsy include the potential for bleeding, pain, and missed biopsy or biopsy of another tissue.

1.6 *Describe:*

- Blood samples will be drawn routinely for the evaluation of safety and toxicity for administration of chemotherapy and to monitor if there is any unexpected side effect from the proglumide. There is always a risk of bruising or discomfort from drawing blood.
- Research blood will be drawn and analyzed for miRNA biomarker panel that predicts response to proglumide and reversal of fibrosis in the TME
- Questionnaire: The McGill pain questionnaire will be obtained at baseline, week 8, 16 and week 24 to gather information on whether proglumide may decrease pain.

7.1 Study design:

Describe and explain the study design.
Overview: Recommended Phase 2 dose (RP2D) of proglumide with standard of care therapy (Gem-NAB-P). The Phase 1 study will be open labeled. And this will be followed by a Phase 2 randomized placebo-controlled trial randomized 1:1 with or without proglumide (or placebo) in combination with GEM-NAB-P for 24 weeks. Biopsy will be obtained in all subjects at baseline and week 8. Research blood will be collected at baseline and every 8 weeks for a miRNA biomarker panel to assess fibrosis.

This is a Phase 2 study with an open labelled lead-in study to approximately treat 30 patients [6 subjects for Lead-in and 24 for Phase 2] enrolled with metastatic pancreatic cancer with combination therapy using standard of care first line therapy with GEM-NAB-P (GEM 1000mg/m^2 IV and NAB-P 125 mg/m^2 given days 1, 8, and 15 every 28 days, and proglumide will be tested at the daily dose of 1200 mg orally given as 400 mg po TID. The lead-in study will determine the safety and tolerability of the 1200 mg

daily dose of proglumide with standard of care GEM-NAB-P. If 0 or 1 of a total of 6 patients at 400mg experiences a DLT, then we will proceed to the Phase 2 randomized trial.

- If ≥ 2 out of 6 patients at 400 mg TID dose experience a DLT, open-labelled lead-in study will be repeated with N=6 subjects using 800 mg daily of (400 mg po BID).
- If 2 or more experience a DLT at 400 mg BID then the study will be terminated

For the Phase 2 portion, patients will be randomized 1:1 to standard of care chemotherapy using gemcitabine (GEM) and nab-paclitaxel (NAB-P) (GEM 1000 mg/m^2 IV and NAB-P 125 mg/m^2 given days 1, 8, and 15 every 28 days) plus placebo or proglumide at the recommended phase 2 dose (RP2D) for proglumide determined in the Lead-in study. Patients will be monitored for safety and toxicity by laboratory blood testing, physical examinations, neurological check, and cardiac monitoring in the Lead-in study. **Dosing of chemotherapy will be managed following USPI recommendations. If dose-related toxicity occurs with proglumide, the medication will be held until the AE resolves. Proglumide will then be re-administered at the prior lower dose.**

The following dose reductions will be made for a drug related toxicity:
Full dose: NAB-P 125 mg/m^2 gemcitabine 1000 mg/m^2
1st reduction: NAB-P 100 mg/m^2 gemcitabine 800 mg/m^2
2nd reduction: NAB-P 75 mg/m^2 gemcitabine 600 mg/m^2
3rd reduction: discontinue both NAB-P and gemcitabine

Dose modifications for neutrophils/platelets:
Day 1: delay doses for ANC < 1500 or Plts < 100k
Day 8: reduce 1 dose level for ANC 500 to < 1000 or Plts 50k to < 75k, withhold doses for ANC < 500 or Plts < 50k
Day 15 (if day 8 doses reduced or given without modification): reduce 1 dose level from day 8 if ANC 500 to < 1000 or Plts 50k to < 75k, withhold doses for ANC < 500 or Plts < 50k
Day 15 (if day 8 doses were withheld): reduce 1 dose level from day 1 if ANC greater than or equal to 1000 or Plts greater than or equal to 75k; reduce 2 dose levels from day 1 if ANC 500 to < 1000 or Plts 50 to < 75k; withhold doses if ANC < 500 or Plt < 50k

Dose modifications for other AEs:
Febrile neutropenia (G3/4): withhold both drugs until fever resolved and ANC 1500 or greater, resume at next lower dose level
Peripheral neuropathy (G3/4): withhold NAB-P until improves to G1 or better, resume at next lower dose level, no dose reduction for gemcitabine
Cutaneous toxicity (G2/3): reduce both drugs to next lower dose level, discontinue treatment if toxicity persists
GI toxicity (G3 mucositis or diarrhea): withhold both drugs until G1 or better, resume at next lower dose level

In Phase 2, a research tumor biopsy will be performed before starting therapy. Every 8 weeks radiographic imaging will be done for assess tumor burden according to SOC. Blood work for safety analysis will be done at 2 weeks, then every 4 weeks, interim vital signs and history is done every 4 weeks. Radiographic imaging and CA19-9 tumor marker will be done every 8 weeks according to SOC. Tumor biopsy will be done at baseline and week 8 only for research purposes to examine the TME. Patients will be treated for 24 weeks or until progression is documented, unacceptable toxicities develop, or treatment is no longer required.

7.1a Schedule of events

Measurements Evaluations	Screen	Treatment																									FU
	Day	Week																									Phone
	–28 to –1	0	1	2	3	4	5	6	7	8	9	10	11	12	13	14	15	16	17	18	19	20	21	22	23	24	28
Dispense Proglumide or Placebo		X				X				X				X				X				X					
Gem-NAB-P		X	X	X		X	X	X		X	X	X		X	X	X		X	X	X		X	X	X			
Informed consent	X																										
Review inclusion / exclusion criteria	X	X																									
Medical history	X	X																									X
Physical examination or interim PE	X	X								X								X								X	

Measurements Evaluations	Screen	Treatment																								FU
	Day	Week																								Phone
	–28 to –1	0	1	2	3	4	5	6	7	8	9	10	11	12	13	14	15	16	17	18	19	20	21	22	23	24	28
Review of concomitant medications	X	X								X								X								X	
Research Blood miRNAs		X								X								X								X	
ECOG	X	X								X								X								X	
Body weight	X	X				X				X				X				X				X				X	
Vital Signs	X	X	X	X		X	X	X		X	X	X		X	X	X		X	X	X		X	X	X		X	
Hematology	X	X	X	X		X	X	X		X	X	X		X	X	X		X	X	X		X	X	X		X	
Chemistry	X	X	X	X		X	X	X		X	X	X		X	X	X		X	X	X		X	X	X		X	
Urinalysis	X	X				X				X								X					X			X	
Serum pregnancy test	X	X	X	X		X	X	X		X	X	X		X	X	X							X			X	
Hepatitis B,C	X																										
CA19-9	X									X								X								X	
AE monitoring						X				X				X				X				X				X	

Measurements Evaluations	Screen	Treatment																								FU
	Day	Week																								Phone
	–28 to –1	0	1	2	3	4	5	6	7	8	9	10	11	12	13	14	15	16	17	18	19	20	21	22	23	24	28
CT / MRI Scan imaging		X								X								X								X	
Pain Questionnaire		X								X																X	
Biopsy	X									X																X	

7.2 Screening phase

Screening procedures will be performed up to 28 days before Day 1, unless otherwise specified. All patients must first read, understand, and sign the IRB/FDA-approved ICF before any study-specific screening procedures are performed. After signing the ICF, completing all screening procedures, and being deemed eligible for entry, patients will be enrolled in the study. Procedures that are performed prior to the signing of the ICF and are considered standard of care may be used as screening assessments if they fall within the 28-day screening window. After signing informed consent, the screening examination will be done including a complete medical history and physical examination (with height, weight, vital signs) by one of the investigators at the Georgetown University Lombardi Comprehensive Cancer Center. Blood tests will be drawn including a hematology panel, a chemistry panel, liver panel, prothrombin time for evaluation of the exclusion criteria. A baseline CA19-9 level will be done for SOC. A serum pregnancy test will be performed in all women of child-bearing potential. Patients will read and sign an informed consent. ECOG score will be determined and disease stage assessed. Patients will undergo a urinalysis. An abdominal CT scan or MRI will be performed unless one was not done within 28days of the screen visit. Treatment must occur within 28 days ± 5 days of the screening visit.

The following procedures will be performed during the Screening Visit:

Informed Consent
Review of eligibility criteria
Medical history and demographics
Complete physical exam
ECOG Performance Status
Vitals signs, weight and height
Review of prior/concomitant medications
Clinical laboratory tests

7.3 Baseline visit

Patients who are deemed eligible for the study during the screening visit will be scheduled for a Baseline visit radiographic imaging and tumor biopsy. The following will be obtained at Baseline or before any administration of the investigational drugs.

Imaging by CT/MRI

A tumor tissue biopsy will be obtained for research purposes for IHC and/ or qRT-PCR and processed for evaluation of the TME. A 7-ml red top blood tube will be collected at baseline for research blood for biomarkers and analysis.

7.4 Treatment phase: (Baseline to week 24)

GEM-NAB-P will be prepared by the pharmacist as an intravenous infusion and given according to SOC protocol. Pre-medications may be given at the discretion of the treating investigator and

typically include dexamethasone 12 mg IV and ondansetron 16 mg IV given 30 minutes prior to chemotherapy.
Vital signs will be obtained. The proglumide dose will be given at 400 mg po TID or 1200 mg orally per day for the lead-in as above for N=6 subjects in an open-labelled fashion. If 2 or more have a DLT the dose will be reduced to 400 mg BID (800 mg daily and repeated with (N=6 subjects) to determine the recommended Phase 2 dose (RP2D). At week-8, and every 8 weeks when imaging is done, a red top tube of blood will be collected for research blood for assays (including cytokines, microRNAs and biomarkers for fibrosis).

In the second Phase of the study all volunteers will be treated with proglumide at the RP2D. Patients will be randomized 1:1 (N=15 per group) to standard of care chemotherapy using gemcitabine (GEM) and nab-paclitaxel (NAB-P) (GEM 1000 mg/m^2 IV and NAB-P 125 mg/m^2 given days 1, 8, and 15 every 28 days) plus placebo or proglumide at the recommended phase 2 dose (RP2D). If the chemotherapy dose is reduced or held for toxicity, proglumide/placebo will still be administered unless deemed inappropriate per the treating investigator (e.g. to be held in instances such as oral intolerance, etc.). Patients will have vital signs and laboratory assessments before each chemotherapy infusion (Days 1, 8, and 15 of each cycle). CBC with differential and CMP will be checked on days 1, 8, and 15 every 28 days; CA19-9 will be checked at baseline and weeks 8, 16, and 24. Every 8 weeks when imaging is done, a 7 ml- red top tube of blood will be collected for research blood for assays (including cytokines, microRNAs and biomarkers for fibrosis).

7.5 End of treatment

End of treatment is defined as the last planned dosing visit within the 24-week dosing period. For patients who discontinue chemotherapy prior to 6 months due to disease progression, intolerance of therapy, patient withdrawing consent, non-compliance, or because the investigator believe the treatment is no longer in the patient's best interest, the end of treatment is considered the last visit where the decision is made to discontinue treatment. However, subjects may continue on gemcitabine with proglumide (or placebo) if NAB-P is discontinued for toxicity outside the DLT period as long as disease remains stable /improved. All required procedures may be completed within ±7 days of the end of treatment visit. Repeat disease assessment is not required if performed within 28 days prior to the end of treatment visit. If an infusion is given at week-24, subjects will return at week 28 for a Safety-closeout visit or they may continue on the chemotherapy but proglumide will not be given beyond week 24.

All patients will be followed for survival until the end of the study regardless of further treatments, or until the sponsor ends the study.

7.6 Description of study procedures

Medical history and physical examination, weight, and vital signs

Findings from medical history (obtained at screening) and physical examination shall be given a baseline grade according to the procedure for AEs. Increases in severity of pre-existing conditions during the study will be considered AEs, with resolution occurring when the grade returns to the pre-study grade or below.

Physical examinations will be performed on study days noted in the Schedule of Assessments.

A complete physical examination will be performed and will include an assessment of the following (as clinically indicated): general appearance, respiratory, cardiovascular, abdomen, skin, head and neck (including ears, eyes, nose and throat), lymph nodes, thyroid, musculoskeletal (including spine and extremities), genital/rectal, and neurological systems and at screening only, height.

Data and Specimen Banking

1.7 All tissue and blood specimens collected in this trial will be de-identified and so no personal identifiers are attached to specimens.

1.8 Tissues from biopsies may be preserved in paraffin blocks or frozen and used in the future for research.

1.9 Samples will be saved in the locked laboratory of Dr. Smith.

Sharing of Results with Subjects

1.10 At the conclusion of the study after all subjects have been treated and tissues analyzed, patients and their referring physicians can be told the results or provided a copy of the manuscript when published.

Study Timelines

1.11 *Describe:*

- The duration of an individual subject's participation in the study is up to week 28; with 24 weeks of therapy using proglumide and a 4-week follow-up safety visit.
- The duration anticipated to enroll all study subjects: 48 months
- The estimated date for the investigators to complete this study (complete primary analyses): 60 months

Inclusion and Exclusion Criteria

11. 1 Inclusion criteria

In this study, subjects are eligible if they have metastatic histological or cytologic confirmation of adenocarcinoma of the pancreas. Both male and female patients of at least 18 years of age will be eligible. Patients must have an ECOG (Eastern Cooperative Oncology Group) Performance status 0-or 1 (Fig 14)(Oken et al., 1982) and an estimated life expectancy of > 3 months. Patients or legal representative must be able to give written consent. Female patients who are menopausal, or of childbearing age that are

EGOG	Description
0	Fully active, able to carry on all pre-disease performance without restrictions
1	Restricted in strenuously physical activity but ambulatory and able to carry out work of a light or sedentary nature, e.g. light housework, office work
2	Ambulatory and capable of all self-care but unable to carry out any work activities. Up and about more than 50% of waking hours.
3	Capable of only limited self-care, confined to bed or chair more than 50% of waking hours.
4	Completely disabled. Cannot carry on self-care. Totally confined to bed or chair.

Fig 14: ECOG Performance Status(Oken et al., 1982).

surgically sterile or practicing birth control (chemical or mechanical) are eligible. All females of child-bearing potential must have a serum pregnancy test upon entry into the study. At least four weeks must pass from significant surgery and the subject must be physically recovered.

For inclusion in the study patients must fulfill all of the following criteria:

1. Written informed consent and any locally-required authorization (e.g., HIPAA in the USA, EU Data Privacy Directive in the EU) obtained from the patient prior to performing any protocol-related procedures, including screening evaluations

2. Age $\geq$ 18 years at time of study entry.

3. Adequate normal organ and marrow function as defined below:

   - Hemoglobin ≥9.0 g/dL

   Absolute neutrophil count (ANC) $\geq$ 1500 per mm^3

   Platelet count ≥100,000 per mm^3)

   Serum bilirubin ≤1.5 x institutional upper limit of normal (ULN).

   AST and ALT ≤2.5 x ULN of normal unless liver metastases are present, in which case it must be ≤5x ULN

   Creatinine clearance (CL) >40 mL/min using the Cockroft-Gault formula.

4. Evidence of post-menopausal status or negative urinary or serum pregnancy test for female pre-menopausal patients. Women will be considered post-menopausal if they have been amenorrheic for 12 months without an alternative medical cause. The following age-specific requirements apply:

   Women <50 years of age would be considered post-menopausal if they have been amenorrheic for 12 months or more following cessation of exogenous hormonal treatments, or if they have luteinizing hormone and follicle-stimulating hormone

levels in the post-menopausal range for the institution or underwent surgical sterilization (bilateral oophorectomy or hysterectomy).

5. Patients must have measurable disease by RECIST v1.1 and disease amenable to serial biopsy.
6. Subjects may not have received prior therapy with Gemcitabine /NAB-P.

11.2 Exclusion criteria

- Subjects with a concurrent malignancy or malignancy within 5years prior to starting study drug, with the exception of adequately treated basal or squamous cell carcinoma, non-melanomatous skin cancer or curatively resected cervical cancer, or localized prostate cancer following definitive therapy.
- Subjects with uncontrolled cardiovascular diseases (congestive heart failure, symptoms of coronary artery disease, cardiac arrhythmias) or have suffered a myocardial infarction in the preceding 6 months).
- Blood anticoagulation that cannot be safely stopped for biopsy.
- Prior anti-tumor therapy with gemcitabine/ NAB-P and not prior anti-cancer therapy within 1-days prior to the biopsy and first dose of study drug.
- Subjects with poorly controlled medical conditions including asthma, chronic obstructive pulmonary disease, diabetes, seizure disorders, known brain metastases, hepatic or renal failure.
- Pregnant or nursing women.
- Men or women of childbearing potential who are unwilling to employ adequate contraception (condoms, diaphragm, birth control pills, injections, intrauterine device [IUD], or abstinence) prior to study entry and for the duration of study participation.
- Any concurrent chemotherapy, IP, biologic, or hormonal therapy for cancer treatment.
- Major surgical procedure (as defined by the Investigator) within 28 days prior to the first dose of IP. Note: Local surgery of isolated lesions for palliative intent is acceptable.
- History of allogenic organ transplantation.
- Uncontrolled intercurrent illness, including but not limited to, ongoing or active infection, symptomatic congestive heart failure, uncontrolled hypertension, unstable angina pectoris, cardiac arrhythmia, interstitial lung disease, serious chronic gastrointestinal conditions associated with diarrhea, or psychiatric illness/social situations that would limit compliance with study requirement, substantially increase

risk of incurring AEs or compromise the ability of the patient to give written informed consent

- Active infection including **tuberculosis** (clinical evaluation that includes clinical history, physical examination and radiographic findings, and TB testing in line with local practice), **hepatitis B** (known positive HBV surface antigen (HBsAg) result), **hepatitis C**, or **human immunodeficiency virus** (positive HIV 1/2 antibodies). Patients with a past or resolved HBV infection (defined as the presence of hepatitis B core antibody [anti-HBc] and absence of HBsAg) are eligible. Patients positive for hepatitis C (HCV) antibody are eligible only if polymerase chain reaction is negative for HCV RNA.
- Receipt of live attenuated vaccine within 30 days prior to the first dose of investigational drug. Note: Patients, if enrolled, should not receive live vaccine whilst receiving IP and up to 30 days after the last dose of IP.
- Female patients who are pregnant or breastfeeding or male or female patients of reproductive potential who are not willing to employ effective birth control from screening to 90 days after the last dose of proglumide therapy.
- Known allergy or hypersensitivity to any of the study drugs or any of the study drug excipients.
- Known allergy or hypersensitivity to proglumide or any excipient.

1.12 Patient is willing and able to comply with the protocol for the duration of the study including undergoing treatment and scheduled visits and examinations including follow up.

Vulnerable Populations

1.13 Vulnerable subjects will not be included in this study including the following:

- Adults unable to consent
- Individuals who are not yet adults (infants, children, teenagers)
- Pregnant women
- Prisoners

Local Number of Subjects

1.14 It is anticipated that 6-9 patients will enroll in the lead-in study and 24 in Phase 2

1.15 We anticipate about a 10% screening failure or drop out. These subjects will be replaced.

Recruitment Methods

1.16 Subjects will be recruited from the Georgetown Lombardi Comprehensive Cancer Center Oncology outpatient clinics and from local referring physicians.

1.17 *Subjects will not receive compensation for their participation.*

Withdrawal of Subjects

1.18 An individual patient will not receive any further investigational product if any of the following occur in the patient in question:

Withdrawal of consent or lost to follow-up

Adverse event that, in the opinion of the investigator or the sponsor, contraindicates further dosing

Patient is determined to have met one or more of the exclusion criteria for study participation at study entry and continuing investigational therapy might constitute a safety risk

Pregnancy or intent to become pregnant

Any AE that meets criteria for discontinuation as defined in protocol

Dose-limiting toxicity if applicable

Patient noncompliance that, in the opinion of the investigator or sponsor, warrants withdrawal; e.g., refusal to adhere to scheduled visits

Initiation of alternative anticancer therapy including another investigational agent

Confirmation of disease progression and investigator determination that the patient is no longer benefiting from treatment with therapy

Patients who are permanently discontinued from receiving investigational product will be followed for safety, including the collection of any protocol-specified blood specimens, unless consent is withdrawn or the patient is lost to follow-up or enrolled in another clinical study. All patients will be followed for survival. Patients who decline to return to the site for evaluations will be offered follow-up by phone every 8 weeks as an alternative

Withdrawal of consent

Patients are free to withdraw from the study at any time (IP and assessments) without prejudice to further treatment.

Patients who withdraw consent for further participation in the study will not receive any further investigational drug or further study observation, with the exception of follow-up for survival, which will continue until the end of the study unless the patient has expressly withdrawn their consent to survival follow-up. Note that the patient may be offered additional tests or tapering of treatment to withdraw safely.

A patient who withdraws consent will always be asked about the reason(s) for withdrawal and the presence of any AE. The Investigator will follow up AEs outside of the clinical study.
If a patient withdraws consent, they will be specifically asked if they are withdrawing consent to:

- all further participation in the study including any further follow up (e.g., survival contact telephone calls)
- withdrawal of consent to the use of their study generated data
- withdrawal to the use of any samples

Risks to Subjects

1.19 Biopsy: The greatest risk involved in this research study involves the tissue biopsy at the onset and again after 8 weeks of therapy. The rationale for the biopsy is to help investigators examine the cancer tissue and how the proglumide therapy will alter the tumor microenvironment by reducing fibrosis or changing the tumor associated immune cells. A separate consent form will be provided by radiology for this procedure. The biopsy will be done using radiology for accuracy. Any time a biopsy is done there is a risk for bleeding, infection and discomfort at the biopsy site.

1.20 Other possible side effects of proglumide: Side effects observed when proglumide may include reduced gastric acidic secretion. Although the amount of acid suppression will not be as profound as that of proton pump inhibitors, side effects of reduced gastric acid may include risk for Clostridium difficile colitis, iron deficiency anemia, and vitamin D malabsorption. At very high doses, there is a possibility that proglumide could affect bowel motility and gall bladder contraction. There have been no other serious toxicities with Proglumide reported on bone marrow, blood chemistry, cardiac, or pulmonary systems.

1.21 As with any experimental drug or new combination therapy, unforeseen side effects may occur.

1.22 Pregnancy: The risks of proglumide to an embryo or fetus are unknown should the subject be or become pregnant; therefore pregnancy prevention is required for this study.

1.23 Blood draw: research blood samples will be drawn at Baseline and every 8 weeks along with SOC samples drawing. There is a small risk of bruising whenever a blood sample is obtained.

Potential Benefits to Subjects

1.24 Potential benefits from this research may be that the investigational drug, proglumide, will change the TME allowing the SOC chemotherapy to work more efficiently.

1.25 Since proglumide was developed as a medication for peptic ulcer disease, those on proglumide may experience less gastroesophageal reflux.

1.26 Proglumide may also block pain receptors on nerves and decrease pain and the requirement for opioid analgesics.

Sample size and statistical evaluation

For analysis of the TME, we found in our pre-clinical murine studies that a sample size of N=10 per group was adequate to show statistical significance compared to controls when treated with proglumide. Images N=10 images per tumor) of fibrosis stains with Masson's trichrome where analyzed by computerized quantitative morphometric densitometry analysis. The mean and medians were compared between 4 treatment groups. When the combination of proglumide and gemcitabine was compared to gemcitabine therapy alone, the analysis showed P <0.001. Since we will have smaller specimens by biopsy, we estimate that a sample size of N=12 with proglumide at the RP2D and N=12 with placebo will be needed. The rationale for using placebo over just SOC controls is that we want the investigators performing the TME analysis and care of patients to be blinded to the treatment to prevent any bias and also we believe more subjects will enroll in the study and consent to the week-8 biopsy if they know they may be taking the investigational drug.

CD8+ cells will be stained by immunohistochemistry as we have described. For immunohistochemistry analysis of TILs, 5 μm fixed tumor sections will be stained with either CD8 Ab (1:75; eBioscience); or Foxp3 Ab (1:30; eBioscience). Slides will scanned on the Core HSTR Leica scanner and immunoreactive cells were counted manually. The total number of immunoreactive lymphocytes per biopsy will be counted. The mean values will be compared between proglumide/ combination therapy arm and Gem-NAB-P treated subjects. In our animal data with N=10 per group, we found marked changes in TILs in the tumors of proglumide/ gemcitabine-treated mice compared to the gemcitabine monotherapy group (P <0.0001); therefore we believe the N=12 will provide similar statistics.

Comparisons of mean numbers number of TILs, TAMs and fibrosis scores in patients' biopsies will be compared using Student's T- test to compare treatment groups to placebo groups.

Survival will be evaluated using the Kaplan Meier survival analysis with Log-Rank analysis. An intent-to-treat analysis will be performed in which the available data from all evaluable patients will be included in the statistical analysis. The parameters of measurement including the histologic fibrosis scores, number of tumor infiltrating immune cells, change in miRNA levels, time to disease progression, the McGill Pain survey, and the safety laboratory tests will be analyzed by the Biostatistician using SAS statistical software (version 8-1) computer assisted program comparing baseline values of each group (placebo versus proglumide). Comparisons will be made from baseline to every 8-week visits and also between each group. A Bonferroni

correction will be applied to correct for the buildup of the type I error rate due to multiple comparisons.

18.1. Data accuracy & protocol compliance

Plans to Assure Accuracy of Data and Protocol Compliance:

The study will be conducted in the Lombardi CCC at Georgetown University. The data for the entire study will be managed under the direction of Dr. Benjamin Weinberg through a secure computer data base system (with anti-viral protection) will automatically back up and double-check all information entered into the database for each study subject.

Monitoring Progress of participants:

Routine monthly forms for the patients' physical examination, concurrent medication, drug accountability (investigational pharmacy) and laboratory reports will be placed in the patients' records and kept in a locked file cabinet in the Clinical Research units of the Lombardi CCC. Laboratory results performed at Georgetown MedStar University hospital will be sent directly to the LCCC record and password protected. The data manager or study coordinator will enter the data from labs and surveys and physical examinations. Data will be collected, managed and monitored through the Data safety Monitoring Board at Lombardi CCC, Georgetown University. All electronic records will be kept in a highly secure database. A patient's identity on these records will be indicated by a case number rather than by name, and the information linking these case numbers with identity will be kept separate from the research records and not transmitted electronically. Only de-identified data will be sent electronically.

Data Management and Confidentiality

- *Describe the data analysis plan, including any statistical procedures or power analysis. Describe the steps that will be taken to secure the data (e.g., training, authorization of access, password protection, encryption, physical controls, certificates of confidentiality, and separation of identifiers and data) during storage, use, and transmission.*

All investigators in the study will be CITI trained and abide by HIPAA compliance. Patients will be assigned a study number by the investigational pharmacy and their research tests results will be entered into an encrypted dual login secure database that is password protected. The treatment of Proglumide versus placebo will be blinded to all the investigators (except the Pharmacist) for the Phase 2 randomized part of this study.

- *Describe any procedures that will be used for quality control of collected data.*

For quality control of the blood assays to assess the biomarkers and fibrosis, RNA will be extracted from samples as they become available and RNA will be converted to cDNA for sample preservation. Samples will be stored in -80deg freezer with an alarm system. All samples will be divided when obtained and marked with patient identifier number and frozen in 2 locations (-80 deg freezer and liquid N2 box, for back-up). Samples will be batched for assays of blood and tissue sections for quality control so samples for comparison are run simultaneously.

- *Describe how data or specimens will be handled study-wide. What information will be included in that data or associated with the specimens?*

Tissue samples will be analyzed for immune cells and fibrosis by immunohistochemistry and Masson's trichrome stain. Some tissue will be used for RNA extraction for qPCR-arrays for fibrosis. Blood samples will be collected and serum aliquoted and frozen in two separate locations and marked with patient de-identified study number. The blood samples will be used to analyze biomarkers for the tumor microenvironment including cytokines and selective microRNAs.

- *Where and how data or specimens will be stored?*

As above, the tissue and blood samples will be stored in both the -80deg freezer in Building D room 338 and also in the liquid N2 freezer in the basement of Lombardi New research building. Some tissue samples will be fixed in paraformaldehyde and paraffin embedded for sectioning. These blocks are kept in a secure file in Dr. Smith's research lab in Building D room 338.

- *How long the data or specimens will be stored?*

For the duration of the 5 years of this study and if an extra no-cost extension is requested for 1 additional year after the completion of the study.

- *Who will have access to the data or specimens?*

Only the research team approved on the grant to conduct the ex-vivo experiments under the supervision of Dr. Smith.

- *Who is responsible for receipt or transmission of the data or specimens*?

Both Drs Weinberg and Smith are responsible for the data from the specimens.

- *How data or specimens will be transported*?

The tissue specimen will be divided after the biopsy and half placed in formalin for fixation and sent to the Histology lab for paraffin embedding. The other half of tissue will be flash frozen in liquid N2 for research analysis. The specimen will be hand delivered by the Study coordinator or Dr. Cao from the research team to the lab for processing. Blood samples collected for research will be spun down in the Clinical Research Unit (CRU) and serum separated and aliquot into 1.8 ml cryotubes and frozen immediately in the -80deg freezer in the CRU after being labeled with patient number.

- *What type of data is being collected, stored, transmitted and shared*?

- Protected Health Information (PHI): N/A
- Personally Identifiable Information (PII): Pharmacy will keep a record of the blinding
- Identified N/A
- Deidentified Only de-identified samples according to patient number will be collected and stored.
- Limited Data Set (LDS): N/A
- Data for vulnerable populations; NA

- Computer software is in place to protect against malware YES

All operating systems and software updates and patches must be applied

- Regularly YES per the Georgetown IT

Data collected is only the minimum data necessary to answer the research

- Question- YES

Codes must be stored separately from the corresponding de-identified

- Data- YES in Pharmacy

Encryption and password protection must be in place on all portable

- devices used to access data-YES

How access to the data is provisioned and approved- Only research staff
Two Factor Authentication for all systems accessing research data YES per Georgetown University login
Qualtrics is the GU approved survey system for which we are licensed-YES
Physical and technical safeguards in place for electronic and paper data

- during collection, storage, transmission and destruction must be in alignment with Georgetown University policies and procedures:

- GU Box is the approved data storage repository for Research Data-YES
- Georgetown University email may not be used to collect, store or transmit sensitive human subject research data or Protected Health Information (PHI)- Agree
    - Data may be shared via GU Box or another secure method such as Secure File Transfer Protocol (SFTP). Data must be encrypted during transmission.- Agree
- Use of non-secured, public wif-fi for this data is prohibited- Agree
- Computer screens must be locked when not in use- Agree
- Data must be securely destroyed at the end of the retention period- Agree

Provisions to Monitor the Data to Ensure the Safety of Subjects

This Data Safety Monitoring Plan (DSMP) describes the specifications for monitoring as required for this project, and is done with adherence to the requirements described in the protocol and Good Clinical Practice.

The DSMP will be reviewed periodically and will be updated as needed. Changes to the DSMP will be made when monitoring specifications change.

The monitor will be the primary contact for the site regarding routine operational study issues. The monitor will contact the site to:

- Discuss the status of the study
- Address protocol questions
- Ask if any serious adverse events, adverse events of special interest, or other safety issues have occurred and if they were reported as required
- Inquire if the site has adequate drug
- Discuss screening/enrollment status and if applicable, encourage further enrollment by suggesting ways for the site to meet enrollment expectations.
- Review issues related to patient information
- Follow up on outstanding documents or action items identified during previous site visits and/or previous communication and assess progress with action plan to resolve

All significant communication with the site will be documented in the Investigator File.

- The AEs related to proglumide therapy and any untoward events will be reviewed at each visit and every 6 months by the Georgetown Lombardi DSMB

Provisions to Protect the Privacy Interests of Subjects

1.27 The investigator must keep all information confidential about the nature of the proposed investigation provided by the sponsor or study monitor to the investigator (with the exception of information required by law or regulations to be disclosed to the IRB, the subject, or the appropriate regulatory authority).

1.28 Subject Anonymity

The anonymity of participating subjects must be maintained. Subjects will be identified by an assigned subject number on CRFs and other documents retrieved from the site or sent to the study monitor, sponsor, regulatory agencies, central laboratories, or blinded reviewers. Documents that identify the subject (e.g., the signed informed consent form) must be maintained in strict confidence by the investigator, except to the extent necessary to allow auditing by the appropriate regulatory authority, the study monitor, or sponsor representatives

Compensation for Research-Related Injury

1.29 If an injury or illness from the study drug, or the procedures required for this study occurs, the reasonable medical expenses required to treat such injury or illness may be paid for by the study sponsor or the charges will be billed to the patients' insurance carrier.

Economic Burden to Subjects

1.1 Patients will be provided a sticker to defray the cost of parking at the Lombardi Georgetown University Hospital but some transportation costs to and from the appointments will not be covered in the study.

Consent Process

1) The informed consent will be obtained by the Co-PIs or the Study Coordinator.

2) In most situations the discussion will take place in the Lombardi Oncology outpatient clinic in a private patient room with the Investigator and /or Study coordinator. In some situations patients may be identified while in the hospital. In this situation one of the investigators will explain the protocol to the patients in a private patient hospital room.

3) After reviewing the consent and procedures with the subject, if they need additional time to decide if they want to participate, the patient may take the consent form home to discuss it with his /her primary care provider or family members. The consent form will then be signed in the presence of witnesses when the subject returns for their procedure.

4) Patients will be reassured that participation in this protocol will not change their standard care and they do not have to participate.

5) Prospective participants will be explained that the blood draw and chemotherapy are SOC and the tissue biopsy procedure and administration of oral proglumide daily is investigational.

Non-English Speaking Subjects

- Spanish –speaking individuals may enroll if eligible and a Spanish speaking non-family member can interpret the consent form and help address questions.

Process to Document Consent in Writing

1.2 We will follow the "SOP: Written Documentation of Consent (HRP-091) process for signing the informed consent

Setting

1.3 The research will be conducted in the Georgetown Lombardi CCC outpatient oncology infusion room. The investigational drug, proglumide will be prepared by the Investigational pharmacist in the Georgetown Clinical Research Unit (CRU) on the 7th floor of the Main Hospital.

Resources Available

- We will use the in place CRU and Lombardi CCC facilities and resources to recruit study subjects as well as the www.clinicaltrials.gov website.

Multi-Site Research

Not applicable

References

Abbruzzese,J.L., Gholson,C.F., Daugherty,K., Larson,E., DuBrow,R., Berlin,R., and Levin,B. (1992). A pilot clinical trial of the cholecystokinin receptor antagonist MK-329 in patients with advanced pancreatic cancer. Pancreas *7,* 165-171.

Apte,M.V., Park,S., Phillips,P.A., Santucci,N., Goldstein,D., Kumar,R.K., Ramm,G.A., Buchler,M., Friess,H., McCarroll,J.A., Keogh,G., Merrett,N., Pirola,R., and Wilson,J.S. (2004). Desmoplastic reaction in pancreatic cancer: role of pancreatic stellate cells. Pancreas *29,* 179-187.

Apte,M.V., Wilson,J.S., Lugea,A., and Pandol,S.J. (2013). A starring role for stellate cells in the pancreatic cancer microenvironment. Gastroenterology *144,* 1210-1219.

Bardram,L., Hilsted,L., and Rehfeld,J.F. (1990). Progastrin expression in mammalian pancreas. Proc. Natl. Acad. Sci. U. S. A *87,* 298-302.

Berna,M.J. and Jensen,R.T. (2007). Role of CCK/gastrin receptors in gastrointestinal/metabolic diseases and results of human studies using gastrin/CCK receptor agonists/antagonists in these diseases. Curr. Top. Med. Chem. *7,* 1211-1231.

Berna,M.J., Seiz,O., Nast,J.F., Benten,D., Blaker,M., Koch,J., Lohse,A.W., and Pace,A. (2010). CCK1 and CCK2 receptors are expressed on pancreatic stellate cells and induce collagen production. J Biol. Chem. *285*, 38905-38914.

Brahmer,J.R., Tykodi,S.S., Chow,L.Q., Hwu,W.J., Topalian,S.L., Hwu,P., Drake,C.G., Camacho,L.H., Kauh,J., Odunsi,K., Pitot,H.C., Hamid,O., Bhatia,S., Martins,R., Eaton,K., Chen,S., Salay,T.M., Alaparthy,S., Grosso,J.F., Korman,A.J., Parker,S.M., Agrawal,S., Goldberg,S.M., Pardoll,D.M., Gupta,A., and Wigginton,J.M. (2012). Safety and activity of anti-PD-L1 antibody in patients with advanced cancer. N. Engl. J Med. *366*, 2455-2465.

Brand,S.J. and Fuller,P.J. (1988). Differential gastrin gene expression in rat gastrointestinal tract and pancreas during neonatal development. J. Biol. Chem. *263,* 5341-5347.

Carriere,C., Young,A.L., Gunn,J.R., Longnecker,D.S., and Korc,M. (2009). Acute pancreatitis markedly accelerates pancreatic cancer progression in mice expressing oncogenic Kras. Biochem. Biophys. Res. Commun. *382*, 561-565.

Chang,R.S., Lotti,V.J., Chen,T.B., and Kunkel,K.A. (1986). Characterization of the binding of [3H]-(+/-)-L-364,718: a new potent, nonpeptide cholecystokinin antagonist radioligand selective for peripheral receptors. Mol. Pharmacol. *30*, 212-217.

Erkan,M., Reiser-Erkan,C., Michalski,C.W., Deucker,S., Sauliunaite,D., Streit,S., Esposito,I., Friess,H., and Kleeff,J. (2009). Cancer-stellate cell interactions perpetuate the hypoxia-fibrosis cycle in pancreatic ductal adenocarcinoma. Neoplasia. *11*, 497-508.

Fang,H. and Declerck,Y.A. (2013). Targeting the tumor microenvironment: from understanding pathways to effective clinical trials. Cancer Res. *73*, 4965-4977.

Farrow,B., Albo,D., and Berger,D.H. (2008). The role of the tumor microenvironment in the progression of pancreatic cancer. J Surg. Res. *149*, 319-328.

Feig,C., Gopinathan,A., Neesse,A., Chan,D.S., Cook,N., and Tuveson,D.A. (2012). The pancreas cancer microenvironment. Clin Cancer Res. *18,* 4266-4276.

Fino,K.K., Matters,G.L., McGovern,C.O., Gilius,E.L., and Smith,J.P. (2012). Downregulation of the CCK-B receptor in pancreatic cancer cells blocks proliferation and promotes apoptosis. Am. J Physiol Gastrointest. Liver Physiol *302*, G1244-G1252.

Gatenby,R.A. and Gillies,R.J. (2004). Why do cancers have high aerobic glycolysis? Nat. Rev. Cancer *4*, 891-899.

Hahne,W.F., Jensen,R.T., Lemp,G.F., and Gardner,J.D. (1981). Proglumide and benzotript: members of a different class of cholecystokinin receptor antagonists. Proc. Natl. Acad. Sci U. S. A *78*, 6304-6308.

Hanahan,D. and Weinberg,R.A. (2011). Hallmarks of cancer: the next generation. Cell *144*, 646-674.

Hidalgo,M. (2010). Pancreatic cancer. N. Engl. J Med. *362*, 1605-1617.

Hingorani,S.R., Petricoin,E.F., Maitra,A., Rajapakse,V., King,C., Jacobetz,M.A., Ross,S., Conrads,T.P., Veenstra,T.D., Hitt,B.A., Kawaguchi,Y., Johann,D., Liotta,L.A., Crawford,H.C., Putt,M.E., Jacks,T., Wright,C.V., Hruban,R.H., Lowy,A.M., and Tuveson,D.A. (2003). Preinvasive and invasive ductal pancreatic cancer and its early detection in the mouse. Cancer Cell *4*, 437-450.

Howatson,A.G. and Carter,D.C. (1985). Pancreatic carcinogenesis-enhancement by cholecystokinin in the hamster-nitrosamine model. Br. J. Cancer *51*, 107-114.

Korc,M. (2007). Pancreatic cancer-associated stroma production. Am. J Surg. *194*, S84-S86.

Leach,D.R., Krummel,M.F., and Allison,J.P. (1996). Enhancement of antitumor immunity by CTLA-4 blockade. Science *271*, 1734-1736.

Longnecker,D.S., Curphey,T.J., Lilja,H.S., French,J.I., and Daniel,D.S. (1980). Carcinogenicity in rats of the nitrosourea amino acid N delta-(N-methyl-N-nitrosocarbamoyl)-L-ornithine. J Environ. Pathol. Toxicol. *4*, 117-129.

Matters,G.L., Cooper,T.K., McGovern,C.O., Gilius,E.L., Liao,J., Barth,B.M., Kester,M., and Smith,J.P. (2014). Cholecystokinin mediates progression and metastasis of pancreatic cancer associated with dietary fat. Dig. Dis. Sci. *59*, 1180-1191.

Matters,G.L., Harms,J.F., McGovern,C.O., Jayakumar,C., Crepin,K., Smith,Z.P., Nelson,M.C., Stock,H., Fenn,C.W., Kaiser,J., Kester,M., and Smith,J.P. (2009). Growth of human pancreatic cancer is inhibited by down-regulation of gastrin gene expression. Pancreas *38*, e151-e161.

McCleane,G.J. (2003). The cholecystokinin antagonist proglumide enhances the analgesic effect of dihydrocodeine. Clin J Pain *19*, 200-201.

Melzack,R. (1975). The McGill Pain Questionnaire: major properties and scoring methods. Pain *1*, 277-299.

Nadella,S., Burks,J., Al-Sabban,A., Inyang,G., Wang,J., Tucker,R.D., Zamanis,M.E., Bukowski,W., Shivapurkar,N., and Smith,J.P. (2018). Dietary Fat Stimulates Pancreatic Cancer Growth and Promotes Fibrosis of the Tumor Microenvironment through the Cholecystokinin Receptor. Am J Physiol Gastrointest. Liver Physiol.

Neesse,A., Michl,P., Frese,K.K., Feig,C., Cook,N., Jacobetz,M.A., Lolkema,M.P., Buchholz,M., Olive,K.P., Gress,T.M., and Tuveson,D.A. (2011). Stromal biology and therapy in pancreatic cancer
1. Gut *60*, 861-868.

Oken,M.M., Creech,R.H., Tormey,D.C., Horton,J., Davis,T.E., McFadden,E.T., and Carbone,P.P. (1982). Toxicity and response criteria of the Eastern Cooperative Oncology Group. Am. J Clin Oncol. *5*, 649-655.

Pardoll,D.M. (2012). The blockade of immune checkpoints in cancer immunotherapy. Nat. Rev. Cancer *12*, 252-264.

Pauletzki,J.G., Xu,Q.W., and Shaffer,E.A. (1995). Inhibition of gallbladder emptying decreases cholesterol saturation in bile in the Richardson ground squirrel. Hepatology *22*, 325-331.

Prasad,N.B., Biankin,A.V., Fukushima,N., Maitra,A., Dhara,S., Elkahloun,A.G., Hruban,R.H., Goggins,M., and Leach,S.D. (2005). Gene expression profiles in pancreatic intraepithelial neoplasia reflect the effects of Hedgehog signaling on pancreatic ductal epithelial cells. Cancer Res. *65*, 1619-1626.

Quante,M., Varga,J., Wang,T.C., and Greten,F.R. (2013). The gastrointestinal tumor microenvironment. Gastroenterology *145*, 63-78.

Rahib,L., Smith,B.D., Aizenberg,R., Rosenzweig,A.B., Fleshman,J.M., and Matrisian,L.M. (2014). Projecting cancer incidence and deaths to 2030: the unexpected burden of thyroid, liver, and pancreas cancers in the United States. Cancer Res. *74*, 2913-2921.

Rai,R., Kim,J.J., Tewari,M., and Shukla,H.S. (2016). Heterogeneous expression of cholecystokinin and gastrin receptor in stomach and pancreatic cancer: An immunohistochemical study. J Cancer Res Ther. *12*, 411-416.

Ryan,D.P., Hong,T.S., and Bardeesy,N. (2014). Pancreatic adenocarcinoma. N. Engl. J Med. *371*, 1039-1049.

Siegel,R., Ma,J., Zou,Z., and Jemal,A. (2014). Cancer statistics, 2014. CA Cancer J Clin *64*, 9-29.

Singh,P., Owlia,A., Espeijo,R., and Dai,B. (1995). Novel gastrin receptors mediate mitogenic effects of gastrin and processing intermediates of gastrin on Swiss 3T3 fibroblasts. Absence of detectable cholecystokinin (CCK)-A and CCK-B receptors. J Biol. Chem. *270*, 8429-8438.

Smith,J.P., Cooper,T.K., McGovern,C.O., Gilius,E.L., Zhong,Q., Liao,J., Molinolo,A.A., Gutkind,J.S., and Matters,G.L. (2014). Cholecystokinin receptor antagonist halts progression of pancreatic cancer precursor lesions and fibrosis in mice. Pancreas *43*, 1050-1059.

Smith,J.P., Fantaskey,A.P., Liu,G., and Zagon,I.S. (1995). Identification of gastrin as a growth peptide in human pancreatic cancer. Am. J. Physiol. *268*, R135-R141.

Smith,J.P., Hamory,M.W., Verderame,M.F., and Zagon,I.S. (1998). Quantitative analysis of gastrin mRNA and peptide in normal and cancerous human pancreas. Int. J. Mol. Med. *2*, 309-315.

Smith,J.P., Harms,J.F., Matters,G.L., McGovern,C.O., Ruggiero,F.M., Liao,J., Fino,K.K., Ortega,E.E., Gilius,E.L., and Phillips,J.A., III (2012). A single nucleotide polymorphism of the cholecystokinin-B receptor predicts risk for pancreatic cancer. Cancer Biol. Ther. *13*, 164-174.

Smith,J.P., Kramer,S.T., and Solomon,T.E. (1991). CCK stimulates growth of six human pancreatic cancer cell lines in serum-free medium. Regul. Pept. *32*, 341-349.

Smith,J.P., Liu,G., Soundararajan,V., McLaughlin,P.J., and Zagon,I.S. (1994). Identification and characterization of CCK-B/gastrin receptors in human pancreatic cancer cell lines. Am. J. Physiol *266*, R277-R283.

Smith,J.P., Rickabaugh,C.A., McLaughlin,P.J., and Zagon,I.S. (1993). Cholecystokinin receptors and PANC-1 human pancreatic cancer cells. Am. J. Physiol *265*, G149-G155.

Smith,J.P., Shih,A., Wu,Y., McLaughlin,P.J., and Zagon,I.S. (1996). Gastrin regulates growth of human pancreatic cancer in a tonic and autocrine fashion. Am. J. Physiol. *270*, R1078-R1084.

Smith,J.P. and Solomon,T.E. (2014). Cholecystokinin and pancreatic cancer: the chicken or the egg? Am. J Physiol Gastrointest. Liver Physiol *306*, G91-G101.

Smith,J.P., Solomon,T.E., Bagheri,S., and Kramer,S. (1990). Cholecystokinin stimulates growth of human pancreatic adenocarcinoma SW-1990. Dig. Dis. Sci. *35*, 1377-1384.

Smith,J.P., Verderame,M.F., McLaughlin,P., Martenis,M., Ballard,E., and Zagon,I.S. (2002). Characterization of the CCK-C (cancer) receptor in human pancreatic cancer. Int. J. Mol. Med. *10*, 689-694.

Smith,J.P., Wang,S., Nadella,S., Jablonski,S.A., and Weiner,L.M. (2017). Cholecystokinin receptor antagonist alters pancreatic cancer microenvironment and increases efficacy of immune checkpoint antibody therapy in mice. Cancer Immunol Immunother.

Strauss,J., Alewine,C., Figg,W.D., and Duffy,A. (2015). Targeting the microenvironment of pancreatic cancer: overcoming treatment barriers and improving local immune responses. Clin Transl. Oncol.

Tamiolakis,D., Venizelos,I., Simopoulos,C., Kotini,A., Jivannakis,T., and Papadopoulos,N. (2004). Does neoplastic gastrin expression remodel the embryonal pattern of the protein? A study in human pancreas. Hepatogastroenterology *51*, 249-252.

Tuveson,D.A., Shaw,A.T., Willis,N.A., Silver,D.P., Jackson,E.L., Chang,S., Mercer,K.L., Grochow,R., Hock,H., Crowley,D., Hingorani,S.R., Zaks,T., King,C., Jacobetz,M.A., Wang,L., Bronson,R.T., Orkin,S.H., DePinho,R.A., and Jacks,T. (2004). Endogenous oncogenic K-ras(G12D) stimulates proliferation and widespread neoplastic and developmental defects. Cancer Cell *5*, 375-387.

Vonderheide,R.H. and Bayne,L.J. (2013). Inflammatory networks and immune surveillance of pancreatic carcinoma. Curr. Opin. Immunol. *25*, 200-205.

Wank,S.A., Harkins,R., Jensen,R.T., Shapira,H., de Weerth,A., and Slattery,T. (1992). Purification, molecular cloning, and functional expression of the cholecystokinin receptor from rat pancreas. Proc. Natl. Acad. Sci. U. S. A *89*, 3125-3129.

Wank,S.A., Pisegna,J.R., and de Weerth,A. (1994). Cholecystokinin receptor family. Molecular cloning, structure, and functional expression in rat, guinea pig, and human. Ann. N. Y. Acad. Sci. *713*, 49-66.

Watkins,L.R., Kinscheck,I.B., and Mayer,D.J. (1984). Potentiation of opiate analgesia and apparent reversal of morphine tolerance by proglumide. Science *224*, 395-396.

Weinberg,D.S., Ruggeri,B., Barber,M.T., Biswas,S., Miknyocki,S., and Waldman,S.A. (1997). Cholecystokinin A and B receptors are differentially expressed in normal pancreas and pancreatic adenocarcinoma. J. Clin. Invest *100*, 597-603.

Weiner,L.M. and Lotze,M.T. (2012). Tumor-cell death, autophagy, and immunity. N. Engl. J Med. *366*, 1156-1158.

Yadav,D. and Lowenfels,A.B. (2013). The epidemiology of pancreatitis and pancreatic cancer. Gastroenterology *144*, 1252-1261.

Zhang,J.G., Cong,B., Li,Q.X., Chen,H.Y., Qin,J., and Fu,L.H. (2011). Cholecystokinin octapeptide regulates lipopolysaccharide-activated B cells co-stimulatory molecule expression and cytokines production in vitro. Immunopharmacol. Immunotoxicol. *33*, 157-163.

Zhang,J.G., Liu,J.X., Jia,X.X., Geng,J., Yu,F., and Cong,B. (2014). Cholecystokinin octapeptide regulates the differentiation and effector cytokine production of CD4 T cells in vitro. Int. Immunopharmacol. *20*, 307-315.

Zheng,L., Xue,J., Jaffee,E.M., and Habtezion,A. (2013). Role of immune cells and immune-based therapies in pancreatitis and pancreatic ductal adenocarcinoma. Gastroenterology *144*, 1230-1240.

Supplementary data

Supplementary Table S1: All adverse events reported during the study; none were due to proglumide.

Body System	Number of Events	Number of Grade 3-5 Events	SAEs
**Blood and lymphatic system disorders**			
Anemia	4	0	0
**Gastrointestinal disorders**			
Abdominal pain	2	1	1
Constipation	1	0	0
Diarrhea	3	0	0
Nausea	3	0	0
**General disorders and administration site conditions**			
Disease Progression	1	1	1
Fatigue	1	1	0
Fever	1	0	0
Flu Like Symptoms	2	0	0
**Hepatobiliary disorders**			
Hepatic Failure	4	2	0
Hepatobiliary disorders - Other, specify: Transaminitis	3	1	0
**Infections and infestations**			
Folliculitis	1	0	0
Paronychia	1	0	0
**Investigations**			
Alanine aminotransferase increased	2	0	0

Body System	Number of Events	Number of Grade 3-5 Events	SAEs
Neutrophil count decreased	8	3	0
Platelet count decreased	5	0	0
Weight Loss	1	0	0
White blood cell decreased	1	0	0
**Metabolism and nutrition disorders**			
Anorexia	1	0	0
Hyponatremia	2	0	0
**Nervous system disorders**			
Dysgeusia	1	0	0
Headache	2	1	0
Peripheral sensory neuropathy	3	0	0
**Respiratory, thoracic and mediastinal disorders**			
Epistaxis	1	0	0
**Skin and subcutaneous tissue disorders**			
Nail discoloration	1	0	0
Rash Maculopapular	1	0	0
Skin hyperpigmentation	1	0	0
**Vascular disorders**			
Thromboembolic event	3	0	0

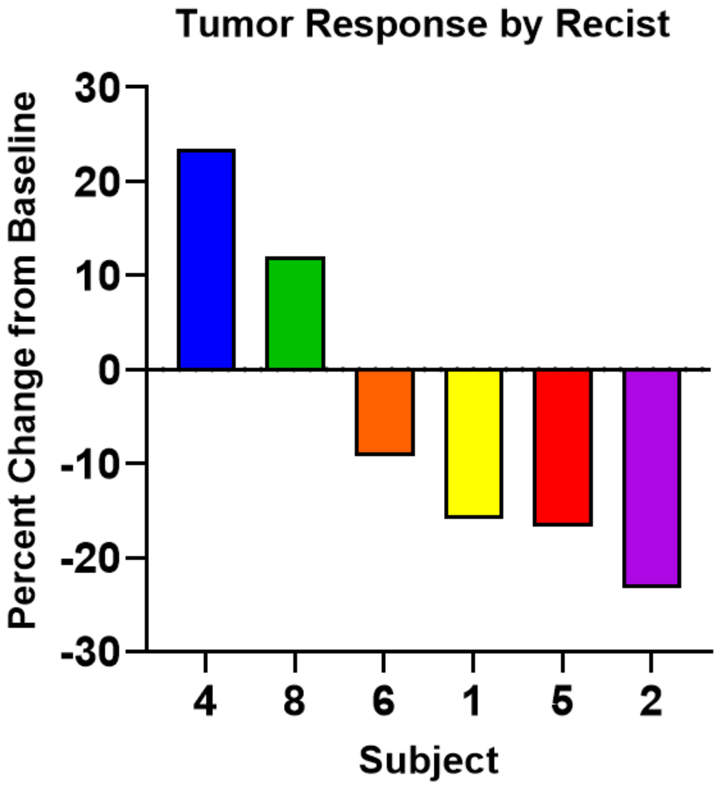

Supplementary Figure S1: Individual patient response according to RECIST criteria.
